# Supplementary material for: Simultaneous Clustering and Estimation of Heterogeneous Graphical Models
Source: J Mach Learn Res. Author manuscript; Available in PMC 2019 Jan 18. (PMC6338433)
Supplement: 1 [file NIHMS996850-supplement-1.pdf]

Online Supplementary

This supplementary contains supporting lemmas and their proofs for the theoretical developments in the main paper.

Appendix A. Proof of Several Lemmas and Selection Consistency

S.I Proof of Lemma 5

The result follows by setting the derivative of $Q(\mathbf{\Theta}'|\mathbf{\Theta})$ with respect to $\boldsymbol{\mu}_k'$ or $\mathbf{\Omega}_k'$ as zero. In particular, solving

$$\frac{\partial Q(\mathbf{\Theta}'|\mathbf{\Theta})}{\partial \boldsymbol{\mu}_k'} = \mathbb{E}[L_{\mathbf{\Theta},k}(\boldsymbol{X})\mathbf{\Omega}_k'(\boldsymbol{X}-\boldsymbol{\mu}_k')] = 0,$$

implies that

$$\arg\max_{\boldsymbol{\mu}_k'} Q(\mathbf{\Theta}'|\mathbf{\Theta}) = \frac{[\mathbf{\Omega}_k']^{-1}\mathbb{E}[L_{\mathbf{\Theta},k}(\boldsymbol{X})\mathbf{\Omega}_k'\boldsymbol{X}]}{\mathbb{E}[L_{\mathbf{\Theta},k}(\boldsymbol{X})]} = \frac{\mathbb{E}[L_{\mathbf{\Theta},k}(\boldsymbol{X})\boldsymbol{X}]}{\mathbb{E}[L_{\mathbf{\Theta},k}(\boldsymbol{X})]}.$$

Similarly, solving

$$\frac{\partial Q(\mathbf{\Theta}'|\mathbf{\Theta})}{\partial \mathbf{\Omega}_k'} = \frac{1}{2}\mathbb{E}[L_{\mathbf{\Theta},k}(\boldsymbol{X})][\mathbf{\Omega}_k']^{-1} - \frac{1}{2}\mathbb{E}[L_{\mathbf{\Theta},k}(\boldsymbol{X})(\boldsymbol{X}-\boldsymbol{\mu}_k')(\boldsymbol{X}-\boldsymbol{\mu}_k')^\top] = 0,$$

implies (19). This ends the proof of Lemma 5. ∎

S.II Proof of Lemma 7

We consider k-th group first

$$\left\|\nabla_{\mathbf{\Theta}_k'} Q(\boldsymbol{\mu}_k^*, \mathbf{\Omega}_k^*|\mathbf{\Theta}^*) - \nabla_{\mathbf{\Theta}_k'} Q(\boldsymbol{\mu}_k^*, \mathbf{\Omega}_k^*|\mathbf{\Theta})\right\|_2 \le \tau \left\|\mathbf{\Theta}-\mathbf{\Theta}^*\right\|_2, \tag{S.1}$$

for any $\mathbf{\Theta} \in \mathbb{B}_\alpha(\mathbf{\Theta}^*)$. Remind that $\mathbf{\Theta}_k' = \mathrm{vec}(\boldsymbol{\mu}_k, \mathbf{\Omega}_k) \in \mathbb{R}^{p^2+p}$. According to the derivation in the proof of Lemma 5, we have

$$\nabla_{\mathbf{\Theta}_k'} Q(\mathbf{\Theta}_k'|\mathbf{\Theta}) = \begin{pmatrix} \mathbb{E}\left[L_{\mathbf{\Theta},k}(\boldsymbol{X})\mathbf{\Omega}_k'(\boldsymbol{X}-\boldsymbol{\mu}_k')\right] \\ \mathrm{vec}\left\{\frac{1}{2}\mathbb{E}[L_{\mathbf{\Theta},k}(\boldsymbol{X})]\mathbf{\Omega}_k'^{-1} - \frac{1}{2}\mathbb{E}[L_{\mathbf{\Theta},k}(\boldsymbol{X})(\boldsymbol{X}-\boldsymbol{\mu}_k')(\boldsymbol{X}-\boldsymbol{\mu}_k')^\top]\right\}^\top \end{pmatrix}.$$

Define $D_L(\mathbf{\Theta}^*, \mathbf{\Theta}) = L_{\mathbf{\Theta}^*,k}(\boldsymbol{X}) - L_{\mathbf{\Theta},k}(\boldsymbol{X})$. Therefore, the square of the left hand side of $(S.1)$ can be simplified to

$$\begin{aligned} & \left\|\nabla_{\mathbf{\Theta}_k'} Q(\boldsymbol{\mu}_k^*, \mathbf{\Omega}_k^*|\mathbf{\Theta}^*) - \nabla_{\mathbf{\Theta}_k'} Q(\boldsymbol{\mu}_k^*, \mathbf{\Omega}_k^*|\mathbf{\Theta})\right\|_2^2 \\ = \; & \underbrace{\left\|\mathbb{E}\left[D_L(\mathbf{\Theta}^*, \mathbf{\Theta})\mathbf{\Omega}_k^*(\boldsymbol{X}-\boldsymbol{\mu}_k^*)\right]\right\|_2^2}_{I} \\ & + \underbrace{\left\|\frac{1}{2}\mathbb{E}[D_L(\mathbf{\Theta}^*, \mathbf{\Theta})\mathbf{\Omega}_k^{*-1} - \frac{1}{2}\mathbb{E}[D_L(\mathbf{\Theta}^*, \mathbf{\Theta})(\boldsymbol{X}-\boldsymbol{\mu}_k^*)(\boldsymbol{X}-\boldsymbol{\mu}_k^*)^\top]\right\|_F^2}_{II}. \end{aligned}$$

If we can show $I \le \tau_1 \|\mathbf{\Theta} - \mathbf{\Theta}^*\|_2^2$ and $II \le \tau_2 \|\mathbf{\Theta} - \mathbf{\Theta}^*\|_2^2$, then we have $\tau = \sqrt{\tau_1 + \tau_2}$ since

$$\left\| \nabla_{\mathbf{\Theta}_k'} Q(\boldsymbol{\mu}_k^*, \mathbf{\Omega}_k^* | \mathbf{\Theta}^*) - \nabla_{\mathbf{\Theta}_k'} Q(\boldsymbol{\mu}_k^*, \mathbf{\Omega}_k^* | \mathbf{\Theta}) \right\|_2 \le \sqrt{\tau_1 + \tau_2} \|\mathbf{\Theta} - \mathbf{\Theta}^*\|_2.$$

Bounding I: We apply Taylor expansion to simplify $D_L(\mathbf{\Theta}^*, \mathbf{\Theta})$. Remind that, by assumption, $\pi_k = 1/K$, and hence we have

$$L_{\mathbf{\Theta},k}(\boldsymbol{X}) = \frac{\pi_k f_k(\boldsymbol{X}; \mathbf{\Theta}_k)}{\sum_{k=1}^K \pi_k f_k(\boldsymbol{X}; \mathbf{\Theta}_k)} = \frac{|\mathbf{\Omega}_k|^{1/2} \exp\left\{-\frac{1}{2}(\boldsymbol{X} - \boldsymbol{\mu}_k)^\top \mathbf{\Omega}_k (\boldsymbol{X} - \boldsymbol{\mu}_k)\right\}}{\sum_{k=1}^K |\mathbf{\Omega}_k|^{1/2} \exp\left\{-\frac{1}{2}(\boldsymbol{X} - \boldsymbol{\mu}_k)^\top \mathbf{\Omega}_k (\boldsymbol{X} - \boldsymbol{\mu}_k)\right\}}.$$

Then, Taylor expansion of $L_{\mathbf{\Theta},k}(\boldsymbol{X})$ around $\mathbf{\Theta}_k^*$ leads to

$$L_{\mathbf{\Theta},k}(\boldsymbol{X}) = L_{\mathbf{\Theta}^*,k}(\boldsymbol{X}) + [\nabla_{\mathbf{\Theta}} L_{\mathbf{\Theta}_t,k}(\boldsymbol{X})]^\top (\mathbf{\Theta} - \mathbf{\Theta}^*), \tag{S.2}$$

where $\mathbf{\Theta}_t = \mathbf{\Theta}^* + t\Delta$ with $t \in [0,1]$ and $\Delta = \mathbf{\Theta} - \mathbf{\Theta}^*$. Here the derivative of $L_{\mathbf{\Theta},k}(\boldsymbol{X})$ with respect to $\mathbf{\Theta} = (\mathbf{\Theta}_1, \ldots, \mathbf{\Theta}_K)$ can be written as

$$\nabla_{\mathbf{\Theta}} L_{\mathbf{\Theta},k}(\boldsymbol{X}) = \left([\nabla_{\mathbf{\Theta}_1} L_{\mathbf{\Theta},k}(\boldsymbol{X})]^\top, \ldots, [\nabla_{\mathbf{\Theta}_K} L_{\mathbf{\Theta},k}(\boldsymbol{X})]^\top \right)^\top, \tag{S.3}$$

where

$$\nabla_{\mathbf{\Theta}_j} L_{\mathbf{\Theta},k}(\boldsymbol{X}) = \begin{cases} -L_{\mathbf{\Theta},k}(\boldsymbol{X}) \cdot L_{\mathbf{\Theta},j}(\boldsymbol{X}) \cdot \delta_{\mathbf{\Theta}_j}(\boldsymbol{X}) \text{ when } j \ne k; \\ L_{\mathbf{\Theta},k}(\boldsymbol{X})[1 - L_{\mathbf{\Theta},k}(\boldsymbol{X})] \cdot \delta_{\mathbf{\Theta}_k}(\boldsymbol{X}) \text{ when } j = k, \end{cases}$$

and, for $j = 1 \ldots, K$, and $\mathbf{\Theta}_j = \mathrm{vec}(\mu_j, \mathbf{\Omega}_j)$,

$$\delta_{\mathbf{\Theta}_j}(\boldsymbol{X}) = \begin{pmatrix} \mathbf{\Omega}_j (\boldsymbol{X} - \boldsymbol{\mu}_j) \\ \frac{1}{2}\mathrm{vec}\left\{ \mathbf{\Omega}_j^{-1} - (\boldsymbol{X} - \boldsymbol{\mu}_j)(\boldsymbol{X} - \boldsymbol{\mu}_j)^\top \right\} \end{pmatrix}.$$

Next we apply this Taylor expansion to bound I. According to $(S.2)$, we have

$$\begin{aligned} I &= \left\| \mathbb{E}\left[\mathbf{\Omega}_k^* (\boldsymbol{X} - \boldsymbol{\mu}_k^*) [\nabla_{\mathbf{\Theta}} L_{\mathbf{\Theta}_t,k}(\boldsymbol{X})]^\top (\mathbf{\Theta} - \mathbf{\Theta}^*) \right] \right\|_2^2 \\ &= \left\| \mathbb{E}\left[\mathbf{\Omega}_k^* (\boldsymbol{X} - \boldsymbol{\mu}_k^*) [\nabla_{\mathbf{\Theta}} L_{\mathbf{\Theta}_t,k}(\boldsymbol{X})]^\top \right] \right\|_2^2 \cdot \|\mathbf{\Theta} - \mathbf{\Theta}^*\|_2^2 \\ &\le \underbrace{\sup_{t \in [0,1]} \mathbb{E}\left[\|\mathbf{\Omega}_k^* (\boldsymbol{X} - \boldsymbol{\mu}_k^*)\|_2^2 \cdot \|\nabla_{\mathbf{\Theta}} L_{\mathbf{\Theta}_t,k}(\boldsymbol{X})\|_2^2 \right]}_{\tau_1} \cdot \|\mathbf{\Theta} - \mathbf{\Theta}^*\|_2^2. \end{aligned}$$

By the definition of $\nabla_{\mathbf{\Theta}} L_{\mathbf{\Theta}_t,k}(\boldsymbol{X})$, which equals to (S.3) with $\mathbf{\Theta} = \mathbf{\Theta}_t$, we have

$$\begin{aligned} \|\nabla_{\mathbf{\Theta}} L_{\mathbf{\Theta}_t,k}(\boldsymbol{X})\|_2^2 &= \underbrace{\sum_{j \ne k} [L_{\mathbf{\Theta}_t,k}(\boldsymbol{X}) L_{\mathbf{\Theta}_t,j}(\boldsymbol{X})]^2 \cdot [\delta_{\mathbf{\Theta}_{tj}}(\boldsymbol{X})]^\top \delta_{\mathbf{\Theta}_{tj}}(\boldsymbol{X})}_{A_1} \\ &\quad + \underbrace{\left[L_{\mathbf{\Theta}_t,k}(\boldsymbol{X}) \big(1 - L_{\mathbf{\Theta}_t,k}(\boldsymbol{X})\big) \right]^2 \cdot [\delta_{\mathbf{\Theta}_{tk}}(\boldsymbol{X})]^\top \delta_{\mathbf{\Theta}_{tk}}(\boldsymbol{X})}_{A_2}. \end{aligned}$$

For each $j = 1, \ldots, K$, we define

$$W_j := \sup_{t \in [0,1]} \mathbb{E}\left\{[\delta_{\mathbf{\Theta}_{tj}}(\boldsymbol{X})]^\top \delta_{\mathbf{\Theta}_{tj}}(\boldsymbol{X}) \cdot \|\mathbf{\Omega}_k^*(\boldsymbol{X} - \boldsymbol{\mu}_k^*)\|_2^2\right\}, \tag{S.4}$$

Then

$$\tau_1 \leq \sup_{t \in [0,1]} \mathbb{E}\left[\|\mathbf{\Omega}_k^*(\boldsymbol{X} - \boldsymbol{\mu}_k^*)\|_2^2 (A_1 + A_2)\right]. \tag{S.5}$$

Under Condition 6, it is sufficient to get an upper bound for τ_1,

$$\begin{aligned}
\tau_1 &\leq \sup_{t \in [0,1]} \mathbb{E}\left[\|\mathbf{\Omega}_k^*(\boldsymbol{X} - \boldsymbol{\mu}_k^*)\|_2^2 A_1\right] + \sup_{t \in [0,1]} \mathbb{E}\left[\|\mathbf{\Omega}_k^*(\boldsymbol{X} - \boldsymbol{\mu}_k^*)\|_2^2 A_2\right] \\
&\leq \sum_{j \neq k} \frac{\gamma^2}{24^2(K-1)^2 M_j} \cdot W_j + \left(\frac{\gamma}{24(K-1)\sqrt{M_k}}(K-1)\right)^2 \cdot W_k.
\end{aligned}$$

It implies that

$$\tau_1 \leq \frac{\gamma^2}{288}. \tag{S.6}$$

Bounding II: We can apply similar trick above to bound II. By triangle inequality, we have

$$\begin{aligned}
II &\leq \underbrace{\left\|\frac{1}{2}\mathbb{E}\big[D_L(\mathbf{\Theta}^*, \mathbf{\Theta})\mathbf{\Omega}_k^{*-1}\big]\right\|_F^2}_{II_1} \\
&\quad + \underbrace{\left\|\frac{1}{2}\mathbb{E}\big[D_L(\mathbf{\Theta}^*, \mathbf{\Theta})(\boldsymbol{X} - \boldsymbol{\mu}_k^*)(\boldsymbol{X} - \boldsymbol{\mu}_k^*)^\top\big]\right\|_F^2}_{II_2}.
\end{aligned}$$

Apply Taylor expansion in $(S.2)$, we obtain

$$\begin{aligned}
II_1 &\leq \underbrace{\frac{1}{2}\mathbb{E}\left[\|\nabla_{\mathbf{\Theta}} L_{\mathbf{\Theta}_t,k}(\boldsymbol{X})\|_2^2 \|\mathbf{\Omega}_k^{*-1}\|_F^2\right]}_{\gamma_{21}} \cdot \|\mathbf{\Theta} - \mathbf{\Theta}^*\|_2^2 \\
II_2 &\leq \underbrace{\frac{1}{2}\mathbb{E}\left[\|\nabla_{\mathbf{\Theta}} L_{\mathbf{\Theta}_t,k}(\boldsymbol{X})\|_2^2 \left\|(\boldsymbol{X} - \boldsymbol{\mu}_k^*)(\boldsymbol{X} - \boldsymbol{\mu}_k^*)^\top\right\|_F^2\right]}_{\gamma_{22}} \cdot \|\mathbf{\Theta} - \mathbf{\Theta}^*\|_2^2.
\end{aligned}$$

Analogously to (S.4), we define

$$W_j^{'} := \sup_{t \in [0,1]} \mathbb{E}\left\{[\delta_{\mathbf{\Theta}_{tj}}(\boldsymbol{X})]^\top \delta_{\mathbf{\Theta}_{tj}}(\boldsymbol{X}) \left\|\mathbf{\Omega}_k^{*-1}\right\|_F^2\right\}, \tag{S.7}$$

$$W_j^{''} := \sup_{t \in [0,1]} \mathbb{E}\left\{[\delta_{\mathbf{\Theta}_{tj}}(\boldsymbol{X})]^\top \delta_{\mathbf{\Theta}_{tj}}(\boldsymbol{X}) \left\|(\boldsymbol{X} - \boldsymbol{\mu}_k^*)(\boldsymbol{X} - \boldsymbol{\mu}_k^*)^\top\right\|_F^2\right\}. \tag{S.8}$$

for each $j = 1, \ldots, K$. Under Condition 6, we have that,

$$\tau_{21} < \frac{\gamma^2}{576}, \quad \tau_{22} < \frac{\gamma^2}{576}, \text{ and hence } \tau_2 < \frac{\gamma^2}{288}.$$

This together with (S.6) implies that $\tau = \sqrt{\tau_1 + \tau_2} < \gamma/12$, namely

$$\left\| \nabla_{\boldsymbol{\Theta}_k'} Q(\boldsymbol{\mu}_k^*, \boldsymbol{\Omega}_k^* | \boldsymbol{\Theta}^*) - \nabla_{\boldsymbol{\Theta}_k'} Q(\boldsymbol{\mu}_k^*, \boldsymbol{\Omega}_k^* | \boldsymbol{\Theta}) \right\|_2 \leq \frac{\gamma}{12}.$$

Now we take the summation

$$\sum_{k=1}^{K} \left\| \nabla_{\boldsymbol{\Theta}_k'} Q(\boldsymbol{\mu}_k^*, \boldsymbol{\Omega}_k^* | \boldsymbol{\Theta}^*) - \nabla_{\boldsymbol{\Theta}_k'} Q(\boldsymbol{\mu}_k^*, \boldsymbol{\Omega}_k^* | \boldsymbol{\Theta}) \right\|_2^2 \leq \frac{\gamma}{12} \|\boldsymbol{\Theta} - \boldsymbol{\Theta}^*\|_2, \tag{S.9}$$

for any $\boldsymbol{\Theta} \in \mathbb{B}_\alpha(\boldsymbol{\Theta}^*)$. This ends the proof of Lemma 7. ■

S.III Proof of Lemma 9

In order to compute γ, we consider each $\boldsymbol{\Theta}_k = \{\mu_k, \boldsymbol{\Omega}_k\}$ individually. That means we prove the following part first:

$$Q_n(\boldsymbol{\Theta}_k' | \boldsymbol{\Theta}) - Q_n(\boldsymbol{\Theta}_k^* | \boldsymbol{\Theta}) - \left\langle \nabla Q_n(\boldsymbol{\Theta}_k^* | \boldsymbol{\Theta}), \boldsymbol{\Theta}_k' - \boldsymbol{\Theta}_k^* \right\rangle \leq -\frac{\gamma}{2} \left\| \boldsymbol{\Theta}_k' - \boldsymbol{\Theta}_k^* \right\|_2^2,$$

where $Q_n(\boldsymbol{\Theta}_k | \boldsymbol{\Theta})$ means we set $\boldsymbol{\Theta}_i$ $i \neq k$ to zero.

It is sufficient to compute γ_k in (22). Remind that $\boldsymbol{\Theta}_k' = \text{vec}(\boldsymbol{\mu}_k, \boldsymbol{\Omega}_k) \in \mathbb{R}^{p^2+p}$. Therefore,

$$\nabla_{\boldsymbol{\Theta}_k'} Q_n(\boldsymbol{\Theta}_k' | \boldsymbol{\Theta}) = ([\nabla_{\boldsymbol{\mu}_k'} Q_n(\boldsymbol{\Theta}_k' | \boldsymbol{\Theta})]^\top, [\text{vec}(\nabla_{\boldsymbol{\Omega}_k'} Q_n(\boldsymbol{\Theta}_k' | \boldsymbol{\Theta}))]^\top)^\top, \tag{S.10}$$

with

$$\begin{aligned}
\nabla_{\boldsymbol{\mu}_k'} Q_n(\boldsymbol{\Theta}_k' | \boldsymbol{\Theta}) &= \frac{1}{n} \sum_{i=1}^{n} \left[L_{\boldsymbol{\Theta},k}(\boldsymbol{x}_i) \boldsymbol{\Omega}_k' (\boldsymbol{x}_i - \boldsymbol{\mu}_k') \right] \\
\nabla_{\boldsymbol{\Omega}_k'} Q_n(\boldsymbol{\Theta}_k' | \boldsymbol{\Theta}) &= \frac{1}{2n} \sum_{i=1}^{n} [L_{\boldsymbol{\Theta},k}(\boldsymbol{x}_i)] \boldsymbol{\Omega}_k'^{-1} \\
&- \frac{1}{2n} \sum_{i=1}^{n} [L_{\boldsymbol{\Theta},k}(\boldsymbol{x}_i)(\boldsymbol{x}_i - \boldsymbol{\mu}_k')(\boldsymbol{x}_i - \boldsymbol{\mu}_k')^\top].
\end{aligned}$$

Denote $h(\boldsymbol{\mu}, \boldsymbol{\Omega}) := \frac{1}{2}(\boldsymbol{x}_i - \boldsymbol{\mu})^\top \boldsymbol{\Omega} (\boldsymbol{x}_i - \boldsymbol{\mu})$. According to the definition in (9), we have

$$\begin{aligned}
Q_n(\boldsymbol{\Theta}_k' | \boldsymbol{\Theta}) - Q_n(\boldsymbol{\Theta}_k^* | \boldsymbol{\Theta}) &= \frac{1}{n} \sum_{i=1}^{n} \Big[L_{\boldsymbol{\Theta},k}(\boldsymbol{x}_i) \big\{ \frac{1}{2} \log \det(\boldsymbol{\Omega}_k') \\
&\quad - \frac{1}{2} \log \det(\boldsymbol{\Omega}_k^*) + h(\boldsymbol{\mu}_k^*, \boldsymbol{\Omega}_k^*) - h(\boldsymbol{\mu}_k', \boldsymbol{\Omega}_k') \big\} \Big].
\end{aligned}$$

This together with $(S.10)$ implies that

$$Q_n(\boldsymbol{\Theta}_k' | \boldsymbol{\Theta}) - Q_n(\boldsymbol{\Theta}_k^* | \boldsymbol{\Theta}) - \left\langle \nabla_{\boldsymbol{\Theta}_k'} Q_n(\boldsymbol{\Theta}_k^* | \boldsymbol{\Theta}), \boldsymbol{\Theta}_k' - \boldsymbol{\Theta}_k^* \right\rangle = I + II,$$

where

$$I = \frac{1}{n}\sum_{i=1}^{n}\Big[L_{\mathbf{\Theta},k}(\boldsymbol{x}_i)\Big\{h(\boldsymbol{\mu}_k^*, \mathbf{\Omega}_k^*) - h(\boldsymbol{\mu}_k', \mathbf{\Omega}_k^*)\Big\}\Big]$$
$$-(\boldsymbol{\mu}_k' - \boldsymbol{\mu}_k^*)^\top \nabla_{\boldsymbol{\mu}_k'} Q_n(\mathbf{\Theta}_k^*|\mathbf{\Theta}^{(t)}),$$
$$II = \frac{1}{n}\sum_{i=1}^{n}\Big[L_{\mathbf{\Theta},k}(\boldsymbol{x}_i)\Big\{\frac{1}{2}\log\det(\mathbf{\Omega}_k') - \frac{1}{2}\log\det(\mathbf{\Omega}_k^*)$$
$$+h(\boldsymbol{\mu}_k', \mathbf{\Omega}_k^*) - h(\boldsymbol{\mu}_k', \mathbf{\Omega}_k')\Big\}\Big] - [\mathrm{vec}(\mathbf{\Omega}_k' - \mathbf{\Omega}_k^*)]^\top \nabla_{\mathbf{\Omega}_k'} Q_n(\mathbf{\Theta}_k^*|\mathbf{\Theta}^{(t)}).$$

By a little algebra, we can show that

$$I = -\frac{1}{2n}\sum_{i=1}^{n} L_{\mathbf{\Theta},k}(\boldsymbol{x}_i)(\boldsymbol{\mu}_k' - \boldsymbol{\mu}_k^*)^\top \mathbf{\Omega}_k^* (\boldsymbol{\mu}_k' - \boldsymbol{\mu}_k^*).$$

Due to the positive definiteness of $\mathbf{\Omega}_k^*$, it is shown the following inequality

$$(\boldsymbol{\mu}_k' - \boldsymbol{\mu}_k^*)^\top (\mathbf{\Omega}_k^* - \sigma_{\min}(\mathbf{\Omega}_k^*) I_p)(\boldsymbol{\mu}_k' - \boldsymbol{\mu}_k^*) \geq 0$$

$$(\boldsymbol{\mu}_k' - \boldsymbol{\mu}_k^*)^\top \mathbf{\Omega}_k^* (\boldsymbol{\mu}_k' - \boldsymbol{\mu}_k^*) \geq (\boldsymbol{\mu}_k' - \boldsymbol{\mu}_k^*)^\top \sigma_{\min}(\mathbf{\Omega}_k^*) I_p (\boldsymbol{\mu}_k' - \boldsymbol{\mu}_k^*) \geq \beta_1 \left\|\boldsymbol{\mu}_k' - \boldsymbol{\mu}_k^*\right\|_2^2.$$

Substituting the above bound, it is shown that

$$I \leq -\frac{\beta_1}{2n}\sum_{i=1}^{n} L_{\mathbf{\Theta},k}(\boldsymbol{x}_i)\|\boldsymbol{\mu}_k' - \boldsymbol{\mu}_k^*\|_2^2. \tag{S.11}$$

Therefore, it remains to show that

$$II \leq -\frac{1}{2n}\sum_{i=1}^{n} \frac{L_{\mathbf{\Theta},k}(\boldsymbol{x}_i)}{2(\beta_2 + 2\alpha)^2}\|\mathrm{vec}(\mathbf{\Omega}_k' - \mathbf{\Omega}_k^*)\|_2^2. \tag{S.12}$$

Note that, in order to show $(S.12)$, it is equivalent to deriving the strong concavity parameter of $g(\mathbf{\Omega}_k)$, where

$$g(\mathbf{\Omega}_k) := \frac{1}{n}\sum_{i=1}^{n}\left[L_{\mathbf{\Theta},k}(\boldsymbol{x}_i)\left\{\frac{1}{2}\log\det(\mathbf{\Omega}_k) - h(\boldsymbol{\mu}_k', \mathbf{\Omega}_k)\right\}\right].$$

To see it, finding the strong concavity parameter of $g(\mathbf{\Omega}_k)$ aims to compute ρ_k such that, for any $\mathbf{\Omega}_k', \mathbf{\Omega}_k^* \in \mathcal{B}_\alpha(\mathbf{\Omega}_k^*)$,

$$g(\mathbf{\Omega}_k') - g(\mathbf{\Omega}_k^*) - \left\langle \mathrm{vec}\left(\nabla g(\mathbf{\Omega}_k^*)\right), \mathrm{vec}(\mathbf{\Omega}_k' - \mathbf{\Omega}_k^*)\right\rangle \leq -\rho_k/2 \cdot \|\mathbf{\Omega}_k' - \mathbf{\Omega}_k^*\|_F^2,$$

where the left hand side is exactly II. According to Taylor expansion, we can expand $g(\mathbf{\Omega}_k')$ around $\mathbf{\Omega}_k^*$ and obtain

$$\begin{aligned} g(\mathbf{\Omega}_k') &= g(\mathbf{\Omega}_k^*) + \left\langle \mathrm{vec}(\nabla g(\mathbf{\Omega}_k^*), \mathrm{vec}(\mathbf{\Omega}_k' - \mathbf{\Omega}_k^*)\right\rangle \\ &\quad + \frac{1}{2}\left[\mathrm{vec}(\mathbf{\Omega}_k' - \mathbf{\Omega}_k^*)\right]^\top \nabla^2 g(\mathbf{Z})\left[\mathrm{vec}(\mathbf{\Omega}_k' - \mathbf{\Omega}_k^*)\right], \end{aligned}$$

where $\mathbf{Z} = t\mathbf{\Omega}'_k + (1-t)\mathbf{\Omega}^*_k$ with $t \in [0,1]$. For any two matrices $\mathbf{A}, \mathbf{B}$, we write $\mathbf{A} \succeq \mathbf{B}$ if $\mathbf{A} - \mathbf{B}$ is positive semi-definite. We denote 1_p as the identity matrix with dimension $p \times p$. And $\sigma_i(A)$ is the i-th eigenvalue of matrix A. Therefore, if we can show that $-\nabla^2 g(\mathbf{Z}) \succeq m\, 1_p$, i.e., the minimal eigenvalue value $\sigma_{\min}(-\nabla^2 g(\mathbf{Z})) \geq m$, for some positive $m \in \mathbb{R}$, then we have the strongly concavity parameter $\rho_k = m$. By the definition, we have $\nabla^2 g(\mathbf{\Omega}^*_k) = -\frac{1}{2n}\sum_{i=1}^n L_{\mathbf{\Theta},k}(\boldsymbol{x}_i)[\mathbf{\Omega}^*_k]^{-1} \otimes [\mathbf{\Omega}^*_k]^{-1}$. Denote $\widetilde{\Delta} = \mathbf{\Omega}'_k - \mathbf{\Omega}^*_k$. We obtain

$$-\nabla^2 g(\mathbf{Z}) = \frac{1}{2n}\sum_{i=1}^n L_{\mathbf{\Theta},k}(\boldsymbol{x}_i)\left(\mathbf{\Omega}^*_k + t\widetilde{\Delta}\right)^{-1} \otimes \left(\mathbf{\Omega}^*_k + t\widetilde{\Delta}\right)^{-1}.$$

According to Theorem 4.2.1 2 in Horn and Johnson (1988), for any two matrices $\mathbf{A}, \mathbf{B}$, the minimal eigenvalue value of $\mathbf{A} \otimes \mathbf{B}$ equals the products of the minimal eigenvalue values of $\mathbf{A}$ and $\mathbf{B}$. Therefore, we have $\sigma_{\min}\left(\mathbf{A}^{-1} \otimes \mathbf{A}^{-1}\right) = \left[\sigma_{\min}(\mathbf{A}^{-1})\right]^2 = [\sigma_{\max}(\mathbf{A})]^{-2} = \|\mathbf{A}\|_2^{-2}$, where $\|\mathbf{A}\|_2$ refers to the spectral norm of matrix $\mathbf{A}$. Hence,

$$\begin{aligned}\sigma_{\min}(-\nabla^2 g(\mathbf{Z})) &= \frac{1}{2n}\sum_{i=1}^n L_{\mathbf{\Theta},k}(\boldsymbol{x}_i)\|\mathbf{\Omega}^*_k + t\widetilde{\Delta}\|_2^{-2} \\ &\geq \frac{1}{2n}\sum_{i=1}^n L_{\mathbf{\Theta},k}(\boldsymbol{x}_i)\left[\|\mathbf{\Omega}^*_k\|_2 + \|t\widetilde{\Delta}\|_2\right]^{-2}.\end{aligned}$$

As $\|\mathbf{\Theta}' - \mathbf{\Theta}^*\| \leq 2\alpha$, $\|\mathbf{\Omega}'_k - \mathbf{\Omega}^*_k\|_2 \leq \|\mathbf{\Theta}' - \mathbf{\Theta}^*\|_2 \leq 2\alpha$. Therefore,

$$\begin{aligned}\sigma_{\min}(-\nabla^2 g(\mathbf{Z})) &\geq \frac{1}{2n}\sum_{i=1}^n L_{\mathbf{\Theta},k}(\boldsymbol{x}_i)\left[\|\mathbf{\Omega}^*_k\|_2 + 2\alpha\right]^{-2} \\ &\geq \frac{1}{2n}\sum_{i=1}^n L_{\mathbf{\Theta},k}(\boldsymbol{x}_i)\left(\beta_2 + 2\alpha\right)^{-2},\end{aligned}$$

which implies (S.12). Putting the upper bound of I and II together,

$$I + II \leq -\underbrace{\frac{1}{2n}\sum_{i=1}^n L_{\mathbf{\Theta},k}(\boldsymbol{x}_i)}_{(a)} \cdot \min\left\{\beta_1, \frac{1}{2(\beta_2 + 2\alpha)^2}\right\}\|\mathbf{\Theta}'_k - \mathbf{\Theta}^*_k\|_2^2. \tag{S.13}$$

However, (a) is a random term but we require a non-random strong concavity parameter. Thus a concentration bound will be applied on it. $\{L_{\mathbf{\Theta},k}(\boldsymbol{x}_i), i = 1, \ldots, n\}$ are independent random variables with $0 \leq L_{\mathbf{\Theta},k}(\boldsymbol{x}_i) \leq 1$. After applying a basic Hoeffding's inequality, we have

$$\mathbb{P}\left(\left|\frac{1}{n}\sum_{i=1}^n L_{\mathbf{\Theta},k}(\boldsymbol{x}_i) - \mathbb{E}[L_{\mathbf{\Theta},k}(\boldsymbol{X})]\right| \leq t\right) \geq 1 - 2e^{-2nt^2},$$

which implies

$$\left|\frac{1}{n}\sum_{i=1}^n L_{\mathbf{\Theta},k}(\boldsymbol{x}_i) - \mathbb{E}[L_{\mathbf{\Theta},k}(\boldsymbol{X})]\right| \leq \sqrt{\frac{1}{2}\log\frac{2K}{\delta}}\sqrt{\frac{1}{n}},$$

with probability at least $1-\delta/K$. As $\sqrt{\log(2K/\delta)/2n}=o(1)$, there exists some constant c such that

$$\sqrt{\frac{\log 2K}{2\delta n}}-\mathbb{E}[L_{\boldsymbol{\Theta},k}(\boldsymbol{X})]\le -c,$$

when n is large enough. Then plugging it into (S.13),

$$I+II\le -\frac{1}{2}c\cdot\min\left\{\beta_1,\frac{1}{2(\beta_2+2\alpha)^2}\right\}\|\boldsymbol{\Theta}_k'-\boldsymbol{\Theta}_k^*\|_2^2,$$

with probability at least $1-\delta/K$, where

$$\gamma=c\min\left\{\beta_1,\frac{1}{2(\beta_2+2\alpha)^2}\right\}.$$

Once the individual strong concavity parameter is computed, we can simply take the summation from 1 to K:

$$\sum_{k=1}^K Q_n(\boldsymbol{\Theta}_k'|\boldsymbol{\Theta})-Q_n(\boldsymbol{\Theta}_k^*|\boldsymbol{\Theta})-\left\langle\nabla Q_n(\boldsymbol{\Theta}_k^*|\boldsymbol{\Theta}),\boldsymbol{\Theta}_k'-\boldsymbol{\Theta}_k^*\right\rangle\le -\frac{1}{2}\sum_{k=1}^K\gamma\left\|\boldsymbol{\Theta}_k'-\boldsymbol{\Theta}_k^*\right\|_2^2$$

which implies

$$Q_n(\boldsymbol{\Theta}'|\boldsymbol{\Theta})-Q_n(\boldsymbol{\Theta}^*|\boldsymbol{\Theta})-\left\langle\nabla Q_n(\boldsymbol{\Theta}^*|\boldsymbol{\Theta}),\boldsymbol{\Theta}'-\boldsymbol{\Theta}^*\right\rangle\le -\frac{1}{2}\gamma\left\|\boldsymbol{\Theta}'-\boldsymbol{\Theta}^*\right\|_2^2$$

with probability at least $1-\delta$. This ends the proof of Lemma 9. ■

S.IV A Key Lemma for Proving Corollary 18

The next lemma computes the statistical errors in Condition 10 for our SCAN penalty and provides explicit forms of the corresponding $\varepsilon_1,\varepsilon_2$ and δ_1,δ_2.

Lemma S.1 *Suppose that Condition 16, 17 hold, then Condition 10 is satisfied for SCAN penalty with*

$$\varepsilon_1=(CK\|\boldsymbol{\Omega}^*\|_\infty+C'K^{1.5})\sqrt{\frac{\log p+\log(e/\delta)}{n}},\delta_1=(18K^2+6K)\delta,\tag{S.14}$$

$$\varepsilon_2=C''\sqrt{p}\sqrt{\frac{K^3\left(\log p+\log(e/\delta)\right)}{n}},\delta_2=(8K^2+2K)\delta,\tag{S.15}$$

for some absolute constant $C,C',C''>0$. Here $\|\boldsymbol{\Omega}^*\|_\infty$ is the overall max induced norm defined as $\|\boldsymbol{\Omega}^*\|_\infty=\max_{k\in[K]}\|\boldsymbol{\Omega}_k^*\|_\infty$.

In Lemma S.1, the number of clusters K is allowed to grow with the sample size n and the dimension p. The diverging rate of K controls the convergence probability at each iteration and is upper bounded to ensure that the statistical errors hold with a high probability tending to 1 with a proper choice of δ, e.g., $\delta=1/p$.

Proof of Lemma S.1: For the first part of this proof, we focus on the upper bound of $\|\nabla Q_n(\mathbf{\Theta}^*|\mathbf{\Theta}) - \nabla Q(\mathbf{\Theta}^*|\mathbf{\Theta})\|_{\mathcal{P}^*}$. Recall that

$$\begin{aligned}
\nabla Q_n(\mathbf{\Theta}^*|\mathbf{\Theta}) - \nabla Q(\mathbf{\Theta}^*|\mathbf{\Theta}) &= \begin{pmatrix} \nabla_{\mathbf{\Theta}_1^*} Q_n(\mathbf{\Theta}^*|\mathbf{\Theta}) - \nabla_{\mathbf{\Theta}_1^*} Q(\mathbf{\Theta}^*|\mathbf{\Theta}) \\ \vdots \\ \nabla_{\mathbf{\Theta}_K^*} Q_n(\mathbf{\Theta}^*|\mathbf{\Theta}) - \nabla_{\mathbf{\Theta}_K^*} Q(\mathbf{\Theta}^*|\mathbf{\Theta}) \end{pmatrix} \\
&= \begin{pmatrix} \nabla_{\boldsymbol{\mu}_1^*} Q_n(\mathbf{\Theta}^*|\mathbf{\Theta}) - \nabla_{\boldsymbol{\mu}_1^*} Q(\mathbf{\Theta}^*|\mathbf{\Theta}) \\ \mathrm{vec}\left\{\nabla_{\mathbf{\Omega}_1^*} Q_n(\mathbf{\Theta}^*|\mathbf{\Theta}) - \nabla_{\mathbf{\Omega}_1^*} Q(\mathbf{\Theta}^*|\mathbf{\Theta})\right\}^\top \\ \vdots \\ \nabla_{\boldsymbol{\mu}_K^*} Q_n(\mathbf{\Theta}^*|\mathbf{\Theta}) - \nabla_{\boldsymbol{\mu}_K^*} Q(\mathbf{\Theta}^*|\mathbf{\Theta}) \\ \mathrm{vec}\left\{\nabla_{\mathbf{\Omega}_K^*} Q_n(\mathbf{\Theta}^*|\mathbf{\Theta}) - \nabla_{\mathbf{\Omega}_K^*} Q(\mathbf{\Theta}^*|\mathbf{\Theta})\right\}^\top \end{pmatrix}. \quad \text{(S.16)}
\end{aligned}$$

For simplicity, we define $h_{\mu_k}(\mathbf{\Theta}^*) = \nabla_{\boldsymbol{\mu}_k^*} Q_n(\mathbf{\Theta}^*|\mathbf{\Theta}) - \nabla_{\boldsymbol{\mu}_k^*} Q(\mathbf{\Theta}^*|\mathbf{\Theta})$ and $h_{\mathbf{\Omega}_k^*}(\mathbf{\Theta}^*) = \nabla_{\mathbf{\Omega}_k^*} Q_n(\mathbf{\Theta}^*|\mathbf{\Theta}) - \nabla_{\mathbf{\Omega}_k^*} Q(\mathbf{\Theta}^*|\mathbf{\Theta})$. Then from the definition of dual norm $\mathcal{P}^*$ (31), we can have

$$\begin{aligned}
&\|\nabla Q_n(\mathbf{\Theta}^*|\mathbf{\Theta}) - \nabla Q(\mathbf{\Theta}^*|\mathbf{\Theta})\|_{\mathcal{P}^*} \le M_1 \max_{k\in[K]} \underbrace{\|h_{\mu_k}(\mathbf{\Theta}^*)\|_\infty}_{I} \\
&+ M_2 \max_{k\in[K]} \underbrace{\left\|h_{\mathbf{\Omega}_k^*}(\mathbf{\Theta}^*)\right\|_{\max}}_{II} + M_3 \underbrace{\max_{i,j} \left\|\left[h_{\mathbf{\Omega}_k^*}(\mathbf{\Theta}^*)\right]_{ij}, \ldots, \left[h_{\mathbf{\Omega}_k^*}(\mathbf{\Theta}^*)\right]_{ij}\right\|_2}_{III},
\end{aligned}$$

which are corresponding to the penalty on element-wise cluster means, element-wise precision matrices and group structures of multiple precision matrices, respectively.

Bounding Statistical Error for k-th Cluster Mean: Referring to the proof in Lemma 5,

$$h_{\mu_k^*}(\mathbf{\Theta}^*) = \frac{1}{n}\sum_{i=1}^n L_{\mathbf{\Theta},k}(\boldsymbol{x}_i)\mathbf{\Omega}_k^*(\boldsymbol{x}_i - \boldsymbol{\mu}_k^*) - \mathbb{E}\left[L_{\mathbf{\Theta},k}(\boldsymbol{X})\mathbf{\Omega}_k^*(\boldsymbol{X} - \boldsymbol{\mu}_k^*)\right].$$

Note that $\|\mathbf{\Omega}_k^*\|_\infty$ is a scalar. By using triangle inequality, we simplify I by two parts:

$$\begin{aligned}
I &\le \|\mathbf{\Omega}_k^*\|_\infty \left\|\frac{1}{n}\sum_{i=1}^n L_{\mathbf{\Theta},k}(\boldsymbol{x}_i)(\boldsymbol{x}_i - \boldsymbol{\mu}_k^*) - \mathbb{E}\left[L_{\mathbf{\Theta},k}(\boldsymbol{X})(\boldsymbol{X} - \boldsymbol{\mu}_k^*)\right]\right\|_\infty \\
&\le \|\mathbf{\Omega}_k^*\|_\infty \underbrace{\left\|\frac{1}{n}\sum_{i=1}^n L_{\mathbf{\Theta},k}(\boldsymbol{x}_i)\boldsymbol{x}_i - \mathbb{E}\left[L_{\mathbf{\Theta},k}(\boldsymbol{X})\boldsymbol{X}\right]\right\|_\infty}_{I_1} \\
&\quad + \|\mathbf{\Omega}_k^*\|_\infty \underbrace{\left\|\left(\frac{1}{n}\sum_{i=1}^n L_{\mathbf{\Theta},k}(\boldsymbol{x}_i) - \mathbb{E}\left[L_{\mathbf{\Theta},k}(\boldsymbol{X})\right]\right)\boldsymbol{\mu}_k^*\right\|_\infty}_{I_2}.
\end{aligned}$$

Bounding I_1: Denote

$$\zeta = \frac{1}{n}\sum_{i=1}^n L_{\mathbf{\Theta},k}(\boldsymbol{x}_i)\boldsymbol{x}_i - \mathbb{E}\left[L_{\mathbf{\Theta},k}(\boldsymbol{X})\boldsymbol{X}\right]$$

For $\zeta \in \mathbb{R}^p$, we consider the j-th coordinate ζ_j of ζ

$$\zeta_j = \frac{1}{n}\sum_{i=1}^{n} L_{\boldsymbol{\Theta},k}(\boldsymbol{x}_i)x_{ij} - \mathbb{E}\left[L_{\boldsymbol{\Theta},k}(\boldsymbol{X})X_j\right]. \tag{S.17}$$

We introduce a set of missing data $\{c_i, i = 1, \ldots, n\}$, which are independent copies of random variable c. The pair $(\boldsymbol{x}_i, c_i)$ are the independent copy of $(\boldsymbol{X}, c)$. Here c takes a value from the set $\{1, \ldots, K\}$, where $c = k'$ indicates that $\boldsymbol{X}$ was generated by the k'-th mixture component. In another word, the conditional distribution of $\boldsymbol{X}$ is defined below:

$$\boldsymbol{X}|c = k' \sim \mathcal{N}(\boldsymbol{\mu}^*_{k'}, \boldsymbol{\Sigma}^*_{k'})$$

$$\mathbb{P}(c = k') = \pi_{k'},\ \sum_{k'}^{K}\pi_{k'} = 1.$$

This is the usual choice of missing data in EM approaches to mixture modeling. The quantity $(\boldsymbol{x}_i, c_i)$ is referred to as the completed data. Now by the assumption, the j-th coordinate x_{ij} of $\boldsymbol{x}_i$ can be rewritten as the form below:

$$x_{ij} = \sum_{k'=1}^{K} I\{c_i = k'\}(\mu^*_{k'j} + V_{k'j}),\ j \in [p] \tag{S.18}$$

where $\mu^*_{k'j}$ is the j-th coordinate of the true cluster mean $\boldsymbol{\mu}^*_{k'}$ and $V_{k'j} \sim \mathcal{N}(0, \boldsymbol{\Sigma}^*_{k'jj})$. Plugging (S.18) into (S.17), it suffices to bound ζ_j.

$$\begin{aligned}
|\zeta_j| \ \leq\ & \left|\frac{1}{n}\sum_{i=1}^{n}\sum_{k'=1}^{K} L_{\boldsymbol{\Theta},k}(\boldsymbol{x}_i)I\{c_i = k'\}\mu^*_{k'j} - \mathbb{E}\left[\sum_{k'=1}^{K} L_{\boldsymbol{\Theta},k}(\boldsymbol{X})I\{c = k'\}\mu^*_{k'j}\right]\right| \\
&+ \left|\frac{1}{n}\sum_{i=1}^{n}\sum_{k'=1}^{K} L_{\boldsymbol{\Theta},k}(\boldsymbol{x}_i)I\{c_i = k'\}V^*_{k'j} - \mathbb{E}\left[\sum_{k'=1}^{K} L_{\boldsymbol{\Theta},k}(\boldsymbol{X})I\{c = k'\}V^*_{k'j}\right]\right| \\
\leq\ & \underbrace{\sum_{k'=1}^{K}\left|\frac{1}{n}\sum_{i=1}^{n} L_{\boldsymbol{\Theta},k}(\boldsymbol{x}_i)I\{c_i = k'\}\mu^*_{k'j} - \mathbb{E}\Big[L_{\boldsymbol{\Theta},k}(\boldsymbol{X})I\{c = k'\}\mu^*_{k'j}\Big]\right|}_{\zeta_{j_1}} \\
&+ \underbrace{\sum_{k'=1}^{K}\left|\frac{1}{n}\sum_{i=1}^{n} L_{\boldsymbol{\Theta},k}(\boldsymbol{x}_i)I\{c_i = k'\}V^*_{k'j} - \mathbb{E}\Big[L_{\boldsymbol{\Theta},k}(\boldsymbol{X})I\{c = k'\}V^*_{k'j}\Big]\right|}_{\zeta_{j_2}}.
\end{aligned}$$

We bound ζ_{j_1} first. Based on the fact that $|L_{\boldsymbol{\Theta},k}(\boldsymbol{x}_i)I\{c_i = k'\}\mu^*_{k'j}| \leq |\mu^*_{k'j}| \leq \|\boldsymbol{\mu}^*_{k'}\|_\infty$ almost surely it can show that $L_{\boldsymbol{\Theta},k}(\boldsymbol{x}_i)I\{c_i = k'\}\mu^*_{k'j}$ is a sub-gaussian random variable with norm $\|\boldsymbol{\mu}^*_{k'}\|_\infty$. Following the Example 5.8 in Vershynin (2012), $\|L_{\boldsymbol{\Theta},k}(\boldsymbol{x}_i)I\{c_i = k'\}\mu^*_{k'j}\|_{\psi_2} \leq \|\boldsymbol{\mu}^*_{k'}\|_\infty$ where $\|\cdot\|_{\psi_2}$ is defined as sub-Gaussian norm. According to supporting Lemma S.5

$$\left\|L_{\boldsymbol{\Theta},k}(\boldsymbol{x}_i)I\{c_i = k'\}\mu^*_{k'j} - \mathbb{E}\left[L_{\boldsymbol{\Theta},k}(\boldsymbol{X})I\{c = k'\}\mu^*_{k'j}\right]\right\|_{\psi_2} \leq 2\left\|\boldsymbol{\mu}^*_{k'}\right\|_\infty.$$

The standard concentration result in supporting Lemma S.6 yields that for every $t \geq 0$ and some constant D_1,

$$\mathbb{P}\left(|\zeta_{j_1}| \geq t\right) \leq e \exp\left(-\frac{D_1 n t^2}{4\|\boldsymbol{\mu}^*_{k'}\|^2_\infty}\right),$$

which implies that, with probability at least $1-\delta$,

$$|\zeta_{j_1}| \leq \sqrt{\frac{4}{D_1}}\|\boldsymbol{\mu}^*_{k'}\|_\infty \sqrt{\frac{\log(e/\delta)}{n}}. \tag{S.19}$$

Now we start to bound ζ_{j_2}. The fact that $L_{\boldsymbol{\Theta},k}(\boldsymbol{x}_i)I\{c_i = k'\} \leq 1$ shows that it is a sub-gaussian random variable with norm $\|L_{\boldsymbol{\Theta},k}(\boldsymbol{x}_i)I\{c_i = k'\}\|_{\psi_2} \leq 1$. $V^*_{k'j}$ is a Gaussian random variable so that it is also a sub-gaussian random variable with norm $\|V^*_{k'j}\|_{\psi_2} \leq (\|\boldsymbol{\Sigma}^*_{k'}\|_{\max})^{1/2}$. Then using the result in supporting Lemma S.4, $L_{\boldsymbol{\Theta},k}(\boldsymbol{x}_i)I\{c_i = k'\}V^*_{k'j}$ is sub-exponential random variable. Moreover, there exists constant D_2 such that

$$\left\|L_{\boldsymbol{\Theta},k}(\boldsymbol{x}_i)I\{c_i = k'\}V^*_{k'j}\right\|_{\psi_1} \leq D_2\Big(\|\boldsymbol{\Sigma}^*_{k'}\|_{\max}\Big)^{1/2}.$$

Supporting lemma S.5 implies

$$\left\|L_{\boldsymbol{\Theta},k}(\boldsymbol{x}_i)I\{c_i = k'\}V^*_{k'j} - \mathbb{E}\left[L_{\boldsymbol{\Theta},k}(\boldsymbol{X})I\{c = k'\}V^*_{k'j}\right]\right\|_{\psi_1} \leq 2D_2\Big(\|\boldsymbol{\Sigma}^*_{k'}\|_{\max}\Big)^{1/2}.$$

Following the concentration inequality of sub-exponential random variables in supporting Lemma S.7, there exists some constant D_3 such that the following inequality

$$\mathbb{P}\Big(|\zeta_{j_2}| \geq t\Big) \leq 2\exp\left(-D_3 \min\left\{\frac{t^2}{4D_2^2\|\boldsymbol{\Sigma}^*_{k'}\|_{\max}}, \frac{t}{2D_2(\|\boldsymbol{\Sigma}^*_{k'}\|_{\max})^{1/2}}\right\} n\right),$$

holds every $t \geq 0$. For sufficient small t, it reduces to

$$\mathbb{P}\Big(|\zeta_{j_2}| \geq t\Big) \leq 2\exp\left(-D_3 \frac{nt^2}{4D_2\|\boldsymbol{\Sigma}^*_{k'}\|_{\max}}\right),$$

which implies that

$$|\zeta_{j_2}| \leq \sqrt{\frac{4D_2}{D_3}}(\|\boldsymbol{\Sigma}^*_{k'}\|_{\max})^{1/2}\sqrt{\frac{\log(2/\delta)}{n}}, \tag{S.20}$$

with probability at least $1-\delta$.

Adding (S.19) and (S.20) together, we have

$$\begin{aligned} |\zeta_{j_1}| + |\zeta_{j_2}| &\leq \sqrt{\frac{4}{D_1}}\|\boldsymbol{\mu}^*_{k'}\|_\infty\sqrt{\frac{\log(e/\delta)}{n}} + \sqrt{\frac{4D_2}{D_3}}(\|\boldsymbol{\Sigma}^*_{k'}\|_{\max})^{1/2}\sqrt{\frac{\log(2/\delta)}{n}} \\ &\leq \sqrt{\frac{4}{D}}\left(\|\boldsymbol{\mu}^*_{k'}\|_\infty + (\|\boldsymbol{\Sigma}^*_{k'}\|_{\max})^{1/2}\right)\sqrt{\frac{\log(e/\delta)}{n}}, \end{aligned}$$

by taking $D = \min\{D_1, D_3/D_2\}$, with at least probability $1-2\delta$. Therefore, it's sufficient to bound $|\zeta_j|$ by

$$|\zeta_j| \leq \sqrt{\frac{4}{D}}\sum_{k'=1}^{K}\left(\|\boldsymbol{\mu}^*_{k'}\|_\infty + (\|\boldsymbol{\Sigma}^*_{k'}\|_{\max})^{1/2}\right)\sqrt{\frac{\log(e/\delta)}{n}},$$

with at least probability $1 - 2K\delta$. Taking the union bound over p coordinates, we obtain

$$I_1 \leq \sqrt{\frac{4}{D}} \sum_{k'=1}^{K} \left(\|\boldsymbol{\mu}_{k'}^*\|_\infty + (\|\boldsymbol{\Sigma}_{k'}^*\|_{\max})^{1/2} \right) \sqrt{\frac{\log(e/\delta) + \log p}{n}}, \tag{S.21}$$

with at least probability $1 - 2K\delta$.

Bounding I_2: Recall that

$$I_2 = \left\| \left(\frac{1}{n} \sum_{i=1}^{n} L_{\boldsymbol{\Theta},k}(\boldsymbol{x}_i) - \mathbb{E}\left[L_{\boldsymbol{\Theta},k}(\boldsymbol{X})\right] \right) \boldsymbol{\mu}_k^* \right\|_\infty \leq \left| \frac{1}{n} \sum_{i=1}^{n} L_{\boldsymbol{\Theta},k}(\boldsymbol{x}_i) - \mathbb{E}\left[L_{\boldsymbol{\Theta},k}(\boldsymbol{X})\right] \right| \|\boldsymbol{\mu}_k^*\|_\infty .$$

$\{L_{\boldsymbol{\Theta},k}(\boldsymbol{x}_i) | i = 1, \ldots n\}$ are bounded independent random variables within interval between 0 and 1. Then it follows Hoeffding's inequality in supporting Lemma S.8 that

$$\mathbb{P}\left(\left| \frac{1}{n} \sum_{i=1}^{n} L_{\boldsymbol{\Theta},k}(\boldsymbol{x}_i) - \mathbb{E}[L_{\boldsymbol{\Theta},k}(\boldsymbol{X})] \right| \leq t \right) \geq 1 - 2e^{-2nt^2},$$

which implies

$$\left| \frac{1}{n} \sum_{i=1}^{n} L_{\boldsymbol{\Theta},k}(\boldsymbol{x}_i) - \mathbb{E}[L_{\boldsymbol{\Theta},k}(\boldsymbol{X})] \right| \leq \sqrt{\frac{1}{2} \log \frac{2}{\delta}} \cdot \sqrt{\frac{1}{n}}, \tag{S.22}$$

with probability at least $1 - \delta$. Combining with the reminder term $\|\boldsymbol{\mu}_k^*\|$,

$$I_2 \leq \sqrt{\frac{1}{2} \log \frac{2}{\delta}} \cdot \sqrt{\frac{1}{n}} \|\boldsymbol{\mu}_k^*\|_\infty. \tag{S.23}$$

Note that the bound in (S.21) is $O_P((\log p/n)^{1/2})$ while the bound in (S.23) is $O_P((1/n)^{1/2})$, there exists some constant D_4 such that $I_2 \leq D_4 I_1$. Consequently, we conclude that I is upper bounded by

$$I \quad \leq \quad (1 + D_4) \|\boldsymbol{\Omega}_k^*\|_\infty \sqrt{\frac{4}{D}} \sum_{k'=1}^{K} \left(\|\boldsymbol{\mu}_{k'}^*\|_\infty + (\|\boldsymbol{\Sigma}_{k'}^*\|_{\max})^{1/2} \right) \sqrt{\frac{\log(e/\delta) + \log p}{n}},$$

with probability at least $1 - (2K + 1)\delta$. For simplicity, let

$$\varphi_K = \sum_{k'=1}^{K} \left(\|\boldsymbol{\mu}_{k'}^*\|_\infty + (\|\boldsymbol{\Sigma}_{k'}^*\|_{\max})^{1/2} \right), \; C_1 = \sqrt{\frac{4(1 + D_4)^2}{D}}. \tag{S.24}$$

Applying union bound,

$$\max_{k \in [K]} I \leq C_1 \|\boldsymbol{\Omega}^*\|_\infty \varphi_K \sqrt{\frac{\log p + \log(e/\delta)}{n}}, \tag{S.25}$$

with probability at least $1 - K(2K + 1)\delta$.

Bounding Statistical Error for k-th Precision Matrix: Referring to the proof in Lemma 5,

$$
\begin{aligned}
h_{\mathbf{\Omega}_k^*}(\mathbf{\Theta}^*) &= \frac{1}{2n}\sum_{i=1}^n L_{\mathbf{\Theta},k}(\boldsymbol{x}_i)\mathbf{\Sigma}_k^* - \frac{1}{2n}\sum_{i=1}^n L_{\mathbf{\Theta},k}(\boldsymbol{x}_i)(\boldsymbol{x}_i-\boldsymbol{\mu}_k^*)(\boldsymbol{x}_i-\boldsymbol{\mu}_k^*)^\top \\
&- \frac{1}{2}\mathbb{E}\left[L_{\mathbf{\Theta},k}(\boldsymbol{X})\right]\mathbf{\Sigma}_k^* + \frac{1}{2}\mathbb{E}\left[L_{\mathbf{\Theta},k}(\boldsymbol{X})(\boldsymbol{X}-\boldsymbol{\mu}_k^*)(\boldsymbol{X}-\boldsymbol{\mu}_k^*)^\top\right].
\end{aligned}
$$

Now we get an explicit from for $h_{\mathbf{\Omega}_k^*}(\mathbf{\Theta}^*)$. Then II is decomposed as below:

$$
\begin{aligned}
II &\le \underbrace{\left\|\frac{1}{2}\left(\frac{1}{n}\sum_{i=1}^n L_{\mathbf{\Theta},k}(\boldsymbol{x}_i)\mathbf{\Sigma}_k^* - \mathbb{E}\left[L_{\mathbf{\Theta},k}(\boldsymbol{X})\mathbf{\Sigma}_k^*\right]\right)\right\|_{\max}}_{II_1} \\
&+ \underbrace{\left\|\frac{1}{2}\left(\frac{1}{n}\sum_{i=1}^n L_{\mathbf{\Theta},k}(\boldsymbol{x}_i)(\boldsymbol{x}_i-\boldsymbol{\mu}_k^*)(\boldsymbol{x}_i-\boldsymbol{\mu}_k^*)^\top - \mathbb{E}\left[L_{\mathbf{\Theta},k}(\boldsymbol{X})(\boldsymbol{X}-\boldsymbol{\mu}_k^*)(\boldsymbol{X}-\boldsymbol{\mu}_k^*)^\top\right]\right)\right\|_{\max}}_{II_2}.
\end{aligned}
$$

The first term is easy to deal with: since $\frac{1}{n}\sum_{i=1}^n L_{\mathbf{\Theta},k}(\boldsymbol{x}_i) - \mathbb{E}\left[L_{\mathbf{\Theta},k}(\boldsymbol{X})\right]$ is scalar by the definition of $L_{\mathbf{\Theta},k}(\boldsymbol{X})$ we can pull it out of the norm. Combining with the result in (S.22), the first term is upper bounded by

$$
II_1 \le \|\mathbf{\Sigma}_k^*\|_{\max}\sqrt{\frac{1}{2}\log\frac{2}{\delta}}\cdot\sqrt{\frac{1}{n}}, \tag{S.26}
$$

with probability at least $1-\delta$.

For the second term II_2, it can be decomposed as four following terms:

$$
\begin{aligned}
II_2 &\le \underbrace{\left\|\frac{1}{2}\left(\frac{1}{n}\sum_{i=1}^n L_{\mathbf{\Theta},k}(\boldsymbol{x}_i)\boldsymbol{x}_i\boldsymbol{x}_i^\top - \mathbb{E}\left[L_{\mathbf{\Theta},k}(\boldsymbol{X})\boldsymbol{X}\boldsymbol{X}^\top\right]\right)\right\|_{\max}}_{II_{21}} \\
&+ \underbrace{\left\|\frac{1}{2}\left(\frac{1}{n}\sum_{i=1}^n L_{\mathbf{\Theta},k}(\boldsymbol{x}_i)\boldsymbol{x}_i\boldsymbol{\mu}_k^{*T} - \mathbb{E}\left[L_{\mathbf{\Theta},k}(\boldsymbol{X})\boldsymbol{X}\boldsymbol{\mu}_k^{*\top}\right]\right)\right\|_{\max}}_{II_{22}} \\
&+ \underbrace{\left\|\frac{1}{2}\left(\frac{1}{n}\sum_{i=1}^n L_{\mathbf{\Theta},k}(\boldsymbol{x}_i)\boldsymbol{\mu}_k^*\boldsymbol{x}_i^\top - \mathbb{E}\left[L_{\mathbf{\Theta},k}(\boldsymbol{X})\boldsymbol{\mu}_k^*\boldsymbol{X}^\top\right]\right)\right\|_{\max}}_{II_{23}} \\
&+ \underbrace{\left\|\frac{1}{2}\left(\frac{1}{n}\sum_{i=1}^n L_{\mathbf{\Theta},k}(\boldsymbol{x}_i)\boldsymbol{\mu}_k^*\boldsymbol{\mu}_k^{*\top} - \mathbb{E}\left[L_{\mathbf{\Theta},k}(\boldsymbol{X})\boldsymbol{\mu}_k^*\boldsymbol{\mu}_k^{*\top}\right]\right)\right\|_{\max}}_{II_{24}}.
\end{aligned}
$$

For the bound of II_{22} and II_{23}, we can just simply pull the $\boldsymbol{\mu}_k^*$ out, which implies

$$\begin{aligned} II_{22} &= \left\| \frac{1}{2}\left(\frac{1}{n}\sum_{i=1}^n L_{\boldsymbol{\Theta},k}(\boldsymbol{x}_i)\boldsymbol{x}_i - \mathbb{E}\left[L_{\boldsymbol{\Theta},k}(\boldsymbol{X})\boldsymbol{X}\right]\right)\boldsymbol{\mu}_k^{*\top} \right\|_{\max} \\ &\le \left\| \frac{1}{2}\left(\frac{1}{n}\sum_{i=1}^n L_{\boldsymbol{\Theta},k}(\boldsymbol{x}_i)\boldsymbol{x}_i - \mathbb{E}\left[L_{\boldsymbol{\Theta},k}(\boldsymbol{X})\boldsymbol{X}\right]\right)\right\|_{\infty} \|\boldsymbol{\mu}_k^*\|_\infty \\ &\overset{(a)}{\le} \sqrt{\frac{4}{D}}\|\boldsymbol{\mu}_k^*\|_\infty \varphi_K \sqrt{\frac{\log(e/\delta)+\log p}{n}}, \end{aligned} \tag{S.27}$$

with probability at least $1-2K\delta$, where (a) follows (S.21).

Next we turn to bound II_{21}. Expand $\boldsymbol{x}_i\boldsymbol{x}_i^\top$ to matrix form for convenient use

$$\boldsymbol{x}_i\boldsymbol{x}_i^\top = \begin{pmatrix} x_{i1}x_{i1} & \dots & x_{i1}x_{ip} \\ \vdots & \ddots & \vdots \\ x_{ip}x_{i1} & \dots & x_{ip}x_{ip} \end{pmatrix}.$$

Since we require a matrix max norm here, it suffices to bound II_{21} individually, namely

$$\zeta_{jj'} = \frac{1}{2}\left(\frac{1}{n}\sum_{i=1}^n L_{\boldsymbol{\Theta},k}(\boldsymbol{x}_i)x_{ij}x_{ij'} - \mathbb{E}\left[L_{\boldsymbol{\Theta},k}(\boldsymbol{X})X_jX_{j'}\right]\right).$$

Recall in (S.18) the j-th coordinate of $\boldsymbol{x}_i$ could be expressed as

$$x_{ij} = \sum_{k'=1}^K I\{c_i = k'\}(\mu_{k'j}^* + V_{k'j}).$$

By straightforward algebra,

$$\begin{aligned} x_{ij}x_{ij'} &= \sum_{k'=1}^K I\{c_i = k'\}(\mu_{k'j}^* + V_{k'j}) \cdot \sum_{k'=1}^K I\{c_i = k'\}(\mu_{k'j'}^* + V_{k'j'}) \\ &\overset{(a)}{=} \sum_{k'=1}^K I\{c_i = k'\}^2(\mu_{k'j}^* + V_{k'j})(\mu_{k'j'}^* + V_{k'j'}) \\ &= \sum_{k'=1}^K I\{c_i = k'\}\left(\mu_{k'j}^*\mu_{k'j'}^* + \mu_{k'j}^*V_{k'j'} + V_{k'j}\mu_{k'j'}^* + V_{k'j}V_{k'j'}\right), \end{aligned}$$

where (a) follows the fact that $I\{c_i = k\}I\{c_i = k'\} = 0$ for any $k \neq k'$. Consequently, we divide $\zeta_{jj'}$ into four parts:

$$\zeta_{jj'} = \frac{1}{2}\sum_{k'=1}^K \left(\zeta_{jj'}(\mu_{k'j}^*\mu_{k'j'}^*) + \zeta_{jj'}(\mu_{k'j}^*V_{k'j'}) + \zeta_{jj'}(V_{k'j}\mu_{k'j'}^*) + \zeta_{jj'}(V_{k'j}V_{k'j'})\right),$$

where

$$\begin{aligned} \zeta_{jj'}(\mu_{k'j}^*\mu_{k'j'}^*) &= \frac{1}{n}\sum_{i=1}^n L_{\boldsymbol{\Theta},k}(\boldsymbol{x}_i)I\{c_i = k'\}\mu_{k'j}^*\mu_{k'j'}^* \\ &\quad -\mathbb{E}\left[L_{\boldsymbol{\Theta},k}(\boldsymbol{X})I\{c = k'\}\mu_{k'j}^*\mu_{k'j'}^*\right]. \end{aligned}$$

Taking the supreme over set $[p]$ in terms of p, p',

$$\sup_{j,j'\in[p]} |\zeta_{jj'}| \leq \sum_{k'=1}^{K} \underbrace{\left(\sup_{j,j'\in[p]} |\zeta_{jj'}(\mu^*_{k'j}\mu^*_{k'j'})|\right)}_{(i)} + \sum_{k'=1}^{K} \underbrace{\left(\sup_{j,j'\in[p]} |\zeta_{jj'}(\mu^*_{k'j}V_{k'j'})|\right)}_{(ii)}$$

$$+ \sum_{k'=1}^{K} \underbrace{\left(\sup_{j,j'\in[p]} |\zeta_{jj'}(V_{k'j}\mu^*_{k'j'})|\right)}_{(iii)} + \sum_{k'=1}^{K} \underbrace{\left(\sup_{j,j'\in[p]} |\zeta_{jj'}(V_{k'j}V_{k'j'})|\right)}_{(iv)}.$$

We will bound (i), (ii), (iii) and (iv) sequentially. $L_{\mathbf{\Theta},k}(\boldsymbol{x}_i)I\{c_i = k'\}\mu^*_{k'j}\mu^*_{k'j'}$ is a sub-gaussian random variable with

$$\|L_{\mathbf{\Theta},k}(\boldsymbol{x}_i)I\{c_i = k'\}\mu^*_{k'j}\mu^*_{k'j'}\|_{\psi_2} \leq \|\boldsymbol{\mu}^*_{k'}\|^2_\infty.$$

According to supporting Lemma S.5,

$$\left\|L_{\mathbf{\Theta},k}(\boldsymbol{x}_i)I\{c_i = k'\}\mu^*_{k'j}\mu^*_{k'j'} - \mathbb{E}[L_{\mathbf{\Theta},k}(\boldsymbol{X})I\{c = k'\}\mu^*_{k'j}\mu^*_{k'j'}]\right\|_{\psi_2} \leq 2\|\boldsymbol{\mu}^*_{k'}\|^2_\infty.$$

Applying concentration inequality in supporting Lemma S.6 yields that

$$\mathbb{P}\left(|\zeta_{jj'}(\mu^*_{k'j}\mu^*_{k'j'})| \leq t\right) \geq 1 - e\exp\left(-\frac{D_4 n t^2}{4\|\boldsymbol{\mu}^*_{k'}\|^4_\infty}\right), \tag{S.28}$$

for any $t > 0$ and some constant D_4. After properly choosing t,

$$(i) \leq \sqrt{\frac{4}{D_4}}\|\boldsymbol{\mu}^*_{k'}\|^2_\infty \sqrt{\frac{\log p + \log(e/\delta)}{n}}, \tag{S.29}$$

with probability at least $1-\delta$. Note that both $L_{\mathbf{\Theta},k}(\boldsymbol{x}_i)I\{c_i = k'\}\mu^*_{k'j}V_{k'j'}$ and $L_{\mathbf{\Theta},k}(\boldsymbol{x}_i)I\{c_i = k'\}V_{k'j'}\mu^*_{k'j}$ are sub-exponential random variables with norm $\|\boldsymbol{\mu}^*_{k'}\|_\infty(\|\mathbf{\Sigma}^*_{k'}\|_{\max})^{1/2}$. Similar to the step in (S.20),

$$\left|\zeta_{jj'}(\mu^*_{k'j}V_{k'j'})\right| \leq \sqrt{\frac{4}{D_5}}\left(\|\boldsymbol{\mu}^*_{k'}\|_\infty(\|\mathbf{\Sigma}^*_{k'}\|_{\max})^{1/2}\right)\sqrt{\frac{\log(2/\delta)}{n}},$$

with at least probability $1-\delta$. Taking the union bound, it is shown that

$$(ii), (iii) \leq \sqrt{\frac{4}{D_5}}\left(\|\boldsymbol{\mu}^*_{k'}\|_\infty(\|\mathbf{\Sigma}^*_{k'}\|_{\max})^{1/2}\right)\sqrt{\frac{\log p + \log(2/\delta)}{n}}, \tag{S.30}$$

with probability at least $1-\delta$ for sufficient large n.

Lastly, the fact that both $L_{\mathbf{\Theta},k}(\boldsymbol{x}_i)I\{c_i = k'\}V_{k'j}$ and $V_{k'j'}$ are sub-gaussian random variables implies $L_{\mathbf{\Theta},k}(\boldsymbol{x}_i)I\{c_i = k'\}V_{k'j}V_{k'j'}$ is sub-exponential random variable with parameter $\|\mathbf{\Sigma}^*_{k'}\|_{\max}$. Applying concentration result, there exists some constant D_6 such that the following inequality

$$\mathbb{P}\left(|\zeta_{jj'}(V_{k'j}V_{k'j'})| \geq t\right) \leq 2\exp\left(-\frac{D_6 n t^2}{4\|\mathbf{\Sigma}^*_{k'}\|^2_{\max}}\right),$$

holds for sufficiently small $t > 0$. Therefore,

$$\mathbb{P}\left(\sup_{j,j'\in[p]} |\zeta_{jj'}(V_{k'j}V_{k'j'})| \geq t\right) \leq 2p^2 \exp\left(-\frac{D_6 n t^2}{4\|\boldsymbol{\Sigma}_{k'}^*\|_{\max}^2}\right).$$

When n is sufficiently large, with probability at least $1-\delta$

$$(iv) \leq \sqrt{\frac{4}{D_6}}\|\boldsymbol{\Sigma}_{k'}^*\|_{\max}\sqrt{\frac{2\log p + \log(2/\delta)}{n}}. \tag{S.31}$$

Putting (S.29), (S.30) and (S.31) together and after some adjustments, II_{21} is upper bounded by

$$II_{21} \leq \sqrt{\frac{1}{D_7}} \sum_{k'=1}^{K}\left(\|\boldsymbol{\mu}_{k'}^*\|_\infty + (\|\boldsymbol{\Sigma}_{k'}^*\|_{\max})^{1/2}\right)^2 \sqrt{\frac{2\log p + \log(e/\delta)}{n}},$$

with probability at least $1-4K\delta$. $D_7 = \min(D_4, D_5, D_6)$. For simplicity, we denote

$$\varphi_K' = \sum_{k'=1}^{K}\left(\|\boldsymbol{\mu}_{k'}^*\|_\infty + (\|\boldsymbol{\Sigma}_{k'}^*\|_{\max})^{1/2}\right)^2.$$

Therefore,

$$II_{21} \leq \sqrt{\frac{2}{D_7}}\varphi_K'\sqrt{\frac{\log p + \log(e/\delta)}{n}}, \tag{S.32}$$

with probability at least $1-4K\delta$.

For the last, it remains to bound II_{24}. Recall that

$$\begin{aligned} II_{24} &= \left\|\frac{1}{2}\left(\frac{1}{n}\sum_{i=1}^{n} L_{\boldsymbol{\Theta},k}(\boldsymbol{x}_i)\boldsymbol{\mu}_k^*\boldsymbol{\mu}_k^{*\top} - \mathbb{E}\left[L_{\boldsymbol{\Theta},k}(\boldsymbol{X})\boldsymbol{\mu}_k^*\boldsymbol{\mu}_k^{*\top}\right]\right)\right\|_{\max} \\ &\leq \left|\frac{1}{2}\left(\frac{1}{n}\sum_{i=1}^{n} L_{\boldsymbol{\Theta},k}(\boldsymbol{x}_i) - \mathbb{E}\left[L_{\boldsymbol{\Theta},k}(\boldsymbol{X})\right]\right)\right| \|\boldsymbol{\mu}_k^*\boldsymbol{\mu}_k^{*\top}\|_{\max}. \end{aligned}$$

Applying the result in (S.22), we have

$$II_{24} \leq \|\boldsymbol{\mu}_k^*\boldsymbol{\mu}_k^{*\top}\|_{\max}\sqrt{\frac{1}{2}\log\frac{2}{\delta}}\cdot\sqrt{\frac{1}{n}}, \tag{S.33}$$

with probability at least $1-\delta$.

Putting (S.27), (S.32) and (S.33) together, now we can have a upper bound for II_2.

$$II_2 \leq \sqrt{\frac{1}{D_7}}\left(2\|\boldsymbol{\mu}_k^*\|_\infty\varphi_K + \varphi_K'\right)\sqrt{\frac{\log p + \log(e/\delta)}{n}}, \tag{S.34}$$

for $D_7 < D/2$ with at least probability $1-(8K+1)\delta$. The upper bound in (S.26) is of order $O_P(n^{-1/2})$ while the upper bound in (S.34) is of order $O_P((\log p/n)^{1/2})$. Thus there exists

some constant D_8 such that $II_1 \le D_8 II_2$. Let $C_2 = ((1+D_8)^2/D_7)^{1/2}$. Applying union bound,

$$\max_{k\in[K]} II \quad \le \quad C_2\left(2\|\boldsymbol{\mu}^*\|_\infty \varphi_K + \varphi_K'\right)\sqrt{\frac{\log p + \log(e/\delta)}{n}}, \tag{S.35}$$

with at least probability $1 - K(8K+2)\delta$.

Bound the Group Structure Part of Precision Matrix:

Recall that

$$\begin{aligned}
III &= \max_{i,j}\Big\| \left[\nabla_{\boldsymbol{\Omega}_1^*} Q_n(\boldsymbol{\Theta}^*|\boldsymbol{\Theta}) - \nabla_{\boldsymbol{\Omega}_1^*} Q(\boldsymbol{\Theta}^*|\boldsymbol{\Theta})\right]_{ij}, \\
&\qquad \ldots, \left[\nabla_{\boldsymbol{\Omega}_K^*} Q_n(\boldsymbol{\Theta}^*|\boldsymbol{\Theta}) - \nabla_{\boldsymbol{\Omega}_K^*} Q(\boldsymbol{\Theta}^*|\boldsymbol{\Theta})\right]_{ij}\Big\|_2 \\
&\le \max_{i,j}\sqrt{K}\Big\| \left[\nabla_{\boldsymbol{\Omega}_1^*} Q_n(\boldsymbol{\Theta}^*|\boldsymbol{\Theta}) - \nabla_{\boldsymbol{\Omega}_1^*} Q(\boldsymbol{\Theta}^*|\boldsymbol{\Theta})\right]_{ij}, \\
&\qquad \ldots, \left[\nabla_{\boldsymbol{\Omega}_K^*} Q_n(\boldsymbol{\Theta}^*|\boldsymbol{\Theta}) - \nabla_{\boldsymbol{\Omega}_K^*} Q(\boldsymbol{\Theta}^*|\boldsymbol{\Theta})\right]_{ij}\Big\|_\infty \\
&\le \sqrt{K}\max_{k\in[K]}\Big\|\left[\nabla_{\boldsymbol{\Omega}_k^*} Q_n(\boldsymbol{\Theta}^*|\boldsymbol{\Theta}) - \nabla_{\boldsymbol{\Omega}_k^*} Q(\boldsymbol{\Theta}^*|\boldsymbol{\Theta})\right]\Big\|_{\max}.
\end{aligned}$$

According to the result in (S.35) and applying union bound over $[K]$,

$$\mathbb{P}\left(III \ge C_2\sqrt{K}\left(2\|\boldsymbol{\mu}_k^*\|_\infty\varphi_K + \varphi_K'\right)\sqrt{\frac{\log p + \log(e/\delta)}{n}}\right) \le K(8K+2)\delta.$$

Thus, III is upper bounded by

$$III \quad \le \quad C_2\sqrt{K}\left(2\|\boldsymbol{\mu}^*\|_\infty\varphi_K + \varphi_K'\right)\sqrt{\frac{\log p + \log(e/\delta)}{n}}, \tag{S.36}$$

with at least probability $1 - K(8K+2)\delta$.

Finally, putting the upper bound (S.25), (S.35) and (S.36) together, we have a upper bound for the following statistical error

$$\begin{aligned}
&\left\|\nabla Q_n(\boldsymbol{\Theta}^*|\boldsymbol{\Theta}) - \nabla Q(\boldsymbol{\Theta}^*|\boldsymbol{\Theta})\right\|_{\mathcal{P}^*} \\
\le\;& C\left((\|\boldsymbol{\Omega}^*\|_\infty + (\sqrt{K}+1)\|\boldsymbol{\mu}^*\|_\infty)\varphi_K + 2(\sqrt{K}+1)\varphi_K'\right)\sqrt{\frac{\log p + \log(e/\delta)}{n}},
\end{aligned}$$

with probability at least $1-(18K+6)\delta$, where $C = \max(M_1C_1, M_2C_2, M_3C_3)$. Under regularity Condition 16, $\varphi_K \le (c_1 + c_2^{1/2})K$, $\varphi_K' \le (c_1+c_2^{1/2})^2 K$. Let $C = C(c_1 + c_2^{1/2})$ and $C' = c_1^2 + c_1c_2^{1/2} + 2(c_1+c_2^{1/2})^2$. Consequently, the upper bound for statistical error can be written as:

$$\|\nabla Q_n(\boldsymbol{\Theta}^*|\boldsymbol{\Theta}) - \nabla Q(\boldsymbol{\Theta}^*|\boldsymbol{\Theta})\|_{\mathcal{P}^*} \le \left(CK\|\boldsymbol{\Omega}^*\|_\infty + C'K^{1.5}\right)\sqrt{\frac{\log p + \log(e/\delta)}{n}},$$

with probability at least $1-(18K+6)\delta$. ■

For the second part of Lemma S.1, we are aiming to bound the statistical error arising from the estimation for diagonal term. The definition of $\mathcal{G}$ in (14) implies that $[\nabla Q_n(\mathbf{\Theta}^*|\mathbf{\Theta}) - \nabla Q(\mathbf{\Theta}^*|\mathbf{\Theta})]_{\mathcal{G}}$ is a Kp-dimensional vector. Following the same derivation before, it suffices to have:

$$\begin{aligned} & \left\|[\nabla Q_n(\mathbf{\Theta}^*|\mathbf{\Theta}) - \nabla Q(\mathbf{\Theta}^*|\mathbf{\Theta})]_{\mathcal{G}}\right\|_2 \\ \leq\ & \sqrt{Kp}\left\|[\nabla Q_n(\mathbf{\Theta}^*|\mathbf{\Theta}) - \nabla Q(\mathbf{\Theta}^*|\mathbf{\Theta})]_{\mathcal{G}}\right\|_{\max} \\ \overset{(a)}{\leq}\ & \sqrt{Kp}\cdot C_2\left(2\|\boldsymbol{\mu}^*\|_\infty \varphi_K + \varphi_K'\right)\sqrt{\frac{\log p + \log(e/\delta)}{n}} \\ =\ & \sqrt{K}\cdot C_2\left(2\|\boldsymbol{\mu}^*\|_\infty \varphi_K + \varphi_K'\right)\sqrt{\frac{p(\log p + \log(e/\delta))}{n}}, \end{aligned}$$

with probability at least $1-(8K^2+2K)\delta$ where (a) comes from (S.36). Now combining two parts together, we end the proof of Lemma S.1. ■

S.V Proof of Lemma 22

For any $\mathbf{\Theta} \in \mathcal{M}$,

$$\begin{aligned} \frac{\mathcal{P}(\mathbf{\Theta})}{\|\mathbf{\Theta}\|_2} &= \frac{\mathcal{P}_1(\mathbf{\Theta})}{\|\mathbf{\Theta}\|_2} + \frac{\mathcal{P}_2(\mathbf{\Theta})}{\|\mathbf{\Theta}\|_2} + \frac{\mathcal{P}_3(\mathbf{\Theta})}{\|\mathbf{\Theta}\|_2} \\ &\leq \frac{M_1\sum_{k=1}^K\sum_{j=1}^p|\mu_{kj}|}{\sqrt{\sum_{k=1}^K\|\boldsymbol{\mu}_k\|_2^2}} + \frac{M_2\sum_{k=1}^K\sum_{i\neq j}|\omega_{kij}|}{\sqrt{\sum_{k=1}^K\|\mathbf{\Omega}_k\|_F^2}} + \frac{\sum_{i\neq j}M_3(\sum_{k=1}^K\omega_{kij}^2)^{1/2}}{\sqrt{\sum_{k=1}^K\|\mathbf{\Omega}_k\|_F^2}}. \end{aligned}$$

By Cauchy's inequality, we can have

$$\frac{\mathcal{P}(\mathbf{\Theta}_{\mathcal{M}})}{\|\mathbf{\Theta}_{\mathcal{M}}\|_2} \leq M_1\sqrt{Kd} + M_2\sqrt{Ks} + M_3\sqrt{s}.$$

Recall that d and s are the sparse parameter for a single cluster mean and precision matrix, respectively. This ends the proof of Lemma 22. ■

S.VI Proof of Lemma 24

First we consider each $\mathbf{\Theta}_k = \{\mu_k, \mathbf{\Omega}_k\}$ individually. That means we prove the following part first:

$$Q_n(\mathbf{\Theta}_k^{(1)}|\mathbf{\Theta}^{(t-1)}) - Q_n(\mathbf{\Theta}_k^{(2)}|\mathbf{\Theta}^{(t-1)}) - \left\langle \nabla_{\mathbf{\Theta}_k} Q_n(\mathbf{\Theta}_k^{(2)}|\mathbf{\Theta}^{(t-1)}), \mathbf{\Theta}_k^{(1)} - \mathbf{\Theta}_k^{(2)}\right\rangle \leq 0,$$

where $Q_n(\mathbf{\Theta}_k|\mathbf{\Theta})$ means we set $\mathbf{\Theta}_i$ $i \neq k$ to zero.

Following the same technique we use in the proof of Lemma (9), the decomposition can be made as below:

$$Q_n(\mathbf{\Theta}_k^{(1)}|\mathbf{\Theta}^{(t-1)}) - Q_n(\mathbf{\Theta}_k^{(2)}|\mathbf{\Theta}^{(t-1)}) - \left\langle \nabla_{\mathbf{\Theta}_k} Q_n(\mathbf{\Theta}_k^{(2)}|\mathbf{\Theta}^{(t-1)}), \mathbf{\Theta}_k^{(1)} - \mathbf{\Theta}_k^{(2)}\right\rangle = I + II,$$

where

$$I = \frac{1}{n}\sum_{i=1}^{n}\Big[L_{\mathbf{\Theta},k}(\boldsymbol{x}_i)\Big\{h(\boldsymbol{\mu}_k^{(2)},\mathbf{\Omega}_k^{(2)}) - h(\boldsymbol{\mu}_k^{(1)},\mathbf{\Omega}_k^{(2)})\Big\}\Big]$$
$$-(\boldsymbol{\mu}_k^{(1)}-\boldsymbol{\mu}_k^{(2)})^\top\nabla_{\boldsymbol{\mu}_k}Q_n(\mathbf{\Theta}_k^{(2)}|\mathbf{\Theta}^{(t-1)}),$$
$$II = \frac{1}{n}\sum_{i=1}^{n}\big[L_{\mathbf{\Theta},k}(\boldsymbol{x}_i)\big\{\frac{1}{2}\log\det(\mathbf{\Omega}_k^{(1)}) - \frac{1}{2}\log\det(\mathbf{\Omega}_k^{(2)})$$
$$+h(\boldsymbol{\mu}_k^{(1)},\mathbf{\Omega}_k^{(2)}) - h(\boldsymbol{\mu}_k^{(1)},\mathbf{\Omega}_k^{(1)})\big\}\big] - [\mathrm{vec}(\mathbf{\Omega}_k^{(1)}-\mathbf{\Omega}_k^{(2)})]^\top\nabla_{\mathbf{\Omega}_k}Q_n(\mathbf{\Theta}_k^{(2)}|\mathbf{\Theta}^{(t-1)}).$$

Bounding I: By a little algebra, we can show that

$$I = -\frac{1}{2n}\sum_{i=1}^{n}L_{\mathbf{\Theta},k}(\boldsymbol{x}_i)(\boldsymbol{\mu}_k^{(1)}-\boldsymbol{\mu}_k^{(2)})^\top\mathbf{\Omega}_k^{(2)}(\boldsymbol{\mu}_k^{(1)}-\boldsymbol{\mu}_k^{(2)}).$$

Plugging in $(\mathbf{\Theta}^{(t)}, t^*\mathbf{\Theta}^{(t)} + (1-t^*)\mathbf{\Theta}^*)$, we have

$$I = -\frac{(1-t^*)^2}{2n}\sum_{i=1}^{n}L_{\mathbf{\Theta},k}(\boldsymbol{x}_i)(\boldsymbol{\mu}_k^{(t)}-\boldsymbol{\mu}_k^*)^\top\left(t^*\mathbf{\Omega}_k^{(t)} + (1-t^*)\mathbf{\Omega}_k^*\right)(\boldsymbol{\mu}_k^{(t)}-\boldsymbol{\mu}_k^*).$$

Recall that $\mathbf{\Theta}^{(t)}$ is the solution of the optimization problem (35). The algorithm guarantees that $\mathbf{\Omega}_k^{(t)}$ is positive definite. Thus, from the positive definiteness of $\mathbf{\Omega}_k^{(t)}$ and $\mathbf{\Omega}_k^*$, it is sufficient to show that

$$I \le 0 \quad \text{holds a.s..} \tag{S.37}$$

When plugging in $(\mathbf{\Theta}^*, t^*\mathbf{\Theta}^{(t)} + (1-t^*)\mathbf{\Theta}^*)$, we have the same conclusion.

Bounding II: Define

$$g(\mathbf{\Omega}_k^{(2)}) := \frac{1}{n}\sum_{i=1}^{n}\left[L_{\mathbf{\Theta},k}(\boldsymbol{x}_i)\left\{\frac{1}{2}\log\det(\mathbf{\Omega}_k^{(2)}) - h\left(\boldsymbol{\mu}_k^{(1)},\mathbf{\Omega}_k^{(2)}\right)\right\}\right].$$

We rewrite II as

$$g(\mathbf{\Omega}_k^{(1)}) - g(\mathbf{\Omega}_k^{(2)}) - \left\langle \mathrm{vec}\left(\nabla g(\mathbf{\Omega}_k^{(2)})\right), \mathrm{vec}\left(\mathbf{\Omega}_k^{(1)}-\mathbf{\Omega}_k^{(2)}\right)\right\rangle.$$

According to Taylor expansion, we can expand $g(\mathbf{\Omega}_k^{(1)})$ around $\mathbf{\Omega}_k^{(2)}$ and obtain

$$\begin{aligned} g(\mathbf{\Omega}_k^{(1)}) &= g(\mathbf{\Omega}_k^{(2)}) + \left\langle \mathrm{vec}(\nabla g(\mathbf{\Omega}_k^{(2)}), \mathrm{vec}(\mathbf{\Omega}_k^{(1)}-\mathbf{\Omega}_k^{(2)})\right\rangle \\ &\quad + \frac{1}{2}\left[\mathrm{vec}(\mathbf{\Omega}_k^{(1)}-\mathbf{\Omega}_k^{(2)})\right]^\top\nabla^2 g(\mathbf{Z})\left[\mathrm{vec}(\mathbf{\Omega}_k^{(1)}-\mathbf{\Omega}_k^{(2)})\right], \end{aligned}$$

where $\mathbf{Z} = t\mathbf{\Omega}_k^{(1)} + (1-t)\mathbf{\Omega}_k^{(2)}$ with $t \in [0,1]$. So an equivalent expression for II is given below:

$$II = \frac{1}{2}\left[\mathrm{vec}(\mathbf{\Omega}_k^{(1)}-\mathbf{\Omega}_k^{(2)})\right]^\top\nabla^2 g(\mathbf{Z})\left[\mathrm{vec}(\mathbf{\Omega}_k^{(1)}-\mathbf{\Omega}_k^{(2)})\right].$$

By the definition of function g we construct, the negative Hessian matrix of function g is

$$-\nabla^2 g(\mathbf{Z}) = \frac{1}{2n}\sum_{i=1}^{n} L_{\mathbf{\Theta},k}(\boldsymbol{x}_i)\mathbf{Z}^{-1}\otimes\mathbf{Z}^{-1}.$$

According to the analysis in the proof of Lemma 9, $\sigma_{\min}\left(\mathbf{Z}^{-1}\otimes\mathbf{Z}^{-1}\right) = \left[\sigma_{\min}(\mathbf{Z}^{-1})\right]^2 \geq 0$. Therefore, $\nabla^2 g(\mathbf{Z})$ is a negative semi-definite matrix, which implies that $II \leq 0$ holds a.s. for any pair of points $(\mathbf{\Theta}^{(1)}, \mathbf{\Theta}^{(2)})$. Incorporating with the fact that $I < 0$, it implies that

$$Q_n(\mathbf{\Theta}_k^{(1)}|\mathbf{\Theta}^{(t-1)}) - Q_n(\mathbf{\Theta}_k^{(2)}|\mathbf{\Theta}^{(t-1)}) - \left\langle \nabla_{\mathbf{\Theta}_k} Q_n(\mathbf{\Theta}_k^{(2)}|\mathbf{\Theta}^{(t-1)}), \mathbf{\Theta}_k^{(1)} - \mathbf{\Theta}_k^{(2)}\right\rangle \leq 0,$$

holds a.s. for pair points $(\mathbf{\Theta}^{(t)}, t^*\mathbf{\Theta}^{(t)} + (1-t^*)\mathbf{\Theta}^*)$, $(\mathbf{\Theta}^{(t)}, t^*\mathbf{\Theta}^{(t)} + (1-t^*)\mathbf{\Theta}^*)$. After doing the summation from 1 to K, we finish the proof of Lemma 24. ■

S.VII Variable Selection Consistency

Theorem S.2 *Denote the final precision matrix estimator as $\widetilde{\mathbf{\Omega}}_k$ and the set of its nonzero off-diagonal elements as $\widetilde{\mathcal{V}}_k$. Under minimal signal condition, we have, with probability tending to 1, $\widetilde{\mathcal{V}}_k = \mathcal{V}_k$ for any $k = 1, \ldots, K$.*

Proof: We prove it in two steps. In Step 1, we show that $\widetilde{\mathcal{V}_k} \supset \mathcal{V}_k$, and in Step 2, we show that $\widetilde{\mathcal{V}_k} \subset \mathcal{V}_k$, both with high probability.

Step 1: In order to prove $\widetilde{\mathcal{V}_k} \supset \mathcal{V}_k$, it is sufficient to show that for any $(i,j) \in \mathcal{V}_k$ with any $k = 1, \ldots, K$, $\widetilde{\omega}_{kij} \neq 0$. Note that

$$|\omega_{kij}^{(T)}| \geq |\omega_{kij}^*| - |\omega_{kij}^{(T)} - \omega_{kij}^*| \geq |\omega_{kij}^*| - \sqrt{\sum_{i,j}(\omega_{kij}^{(T)} - \omega_{kij}^*)^2},$$

Moreover,

$$\sqrt{\sum_{i,j}(\omega_{kij}^{(T)} - \omega_{kij}^*)^2} \leq \|\mathbf{\Theta}^{(T)} - \mathbf{\Theta}^*\|_2. \tag{S.38}$$

According to Corollary 18 and minimal signal condition we have

$$|\omega_{kij}^{(T)}| > r_n.$$

Therefore, we see that $\widetilde{\omega}_{kij} \neq 0$, which implies $\widetilde{\mathcal{V}_k} \supset \mathcal{V}_k$.

Step 2: In order to show $\widetilde{\mathcal{V}_k} \subset \mathcal{V}_k$, we need to check that, for any $(i,j) \in \mathcal{V}_k^c$, the estimator $\widetilde{\omega}_{kij} = 0$. Note that, the estimator before the thresholding step satisfies,

$$|\omega_{kij}^{(T)}| = |\omega_{kij}^{(T)} - \omega_{kij}^*| \leq \sqrt{\sum_{i,j}(\omega_{kij}^{(T)} - \omega_{kij}^*)^2}.$$

From (S.38), it is known that $|\omega_{kij}^{(T)}| \leq r_n$. Therefore, the thresholding step will set $\widetilde{\omega}_{kij} = \omega_{kij}^{(T)}\, 1\{|\widehat{\omega}_{kij}| > r_n\} = 0$ with high probability. This ends the proof of Theorem S.2. ■

Appendix B. Updates steps of our SCAN algorithm

S.I Proof of Lemma 2:

The KKT conditions for μ_{kj} to be a maximizer of $Q(\boldsymbol{\Theta}|\boldsymbol{\Theta}^{(t-1)}) - \mathcal{R}(\boldsymbol{\Theta})$ are

$$\frac{1}{n}\sum_{i=1}^{n} L_{\boldsymbol{\Theta}^{(t-1)},k}\Big(\sum_{l=1}^{p}(x_{il}-\mu_{kl})\omega_{klj}\Big) = \lambda_1 \text{sign}(\mu_{kj}), \text{ when } \mu_{kj}\neq 0,$$
$$\left|\frac{1}{n}\sum_{i=1}^{n} L_{\boldsymbol{\Theta}^{(t-1)},k}\Big(\sum_{l=1,l\neq j}^{p}(x_{il}-\mu_{kl})\omega_{klj} + x_{ij}\omega_{kjj}\Big)\right| \leq \lambda_1, \text{ when } \mu_{kj} = 0.$$

Therefore, the update of $\mu_{kj}^{(t)}$ is given as:

$$\text{If } \left|\frac{1}{n}\sum_{i=1}^{n} L_{\boldsymbol{\Theta}^{(t-1)},k}(\boldsymbol{x}_i)\Big(\sum_{l=1,l\neq j}^{p}(x_{il}-\mu_{kl}^{(t-1)})\omega_{klj}^{(t-1)} + x_{ij}\omega_{kjj}^{(t-1)}\Big)\right| \leq \lambda_1,$$

then $\mu_{kj}^{(t)} = 0$; Else

$$\mu_{kj}^{(t)} = \Big(\omega_{kjj}^{(t-1)}\frac{1}{n}\sum_{i=1}^{n} L_{\boldsymbol{\Theta}^{(t-1)},k}(\boldsymbol{x}_i)\Big)^{-1}\Big\{\frac{1}{n}\sum_{i=1}^{n} L_{\boldsymbol{\Theta}^{(t-1)},k}(\boldsymbol{x}_i)\big(\sum_{l=1}^{p} x_{il}\omega_{klj}^{(t-1)}\big) -$$
$$\big(\frac{1}{n}\sum_{i=1}^{n} L_{\boldsymbol{\Theta}^{(t-1)},k}(\boldsymbol{x}_i)\big)\Big(\sum_{l=1}^{p}\mu_{kl}^{(t-1)}\omega_{klj}^{(t-1)} - \mu_{kj}^{(t-1)}\omega_{kjj}^{(t-1)}\Big) - \lambda_1 \text{sign}(\mu_{kj}^{(t-1)})\Big\}$$

Using the definitions of $g_{1,j}(\boldsymbol{x};\boldsymbol{\Theta}_k^{(t-1)})$ and $g_{2,j}(\boldsymbol{x}_i;\boldsymbol{\Theta}_k^{(t-1)})$, we finish the proof of Lemma 2. ■

S.II Proof of Lemma 3:

Recall that in (8)

$$Q_n(\boldsymbol{\Theta}|\boldsymbol{\Theta}^{(t-1)}) := \frac{1}{n}\sum_{i=1}^{n}\sum_{k=1}^{K} L_{\boldsymbol{\Theta}^{(t-1)},k}(\boldsymbol{x}_i)[\log \pi_k + \log f_k(\boldsymbol{x}_i;\boldsymbol{\Theta}_k)] - \mathcal{R}(\boldsymbol{\Theta}),$$

Then,

$$\begin{aligned}
&\max_{\boldsymbol{\Omega}_1,\ldots,\boldsymbol{\Omega}_K} \frac{1}{n}\sum_{i=1}^{n}\sum_{k=1}^{K} L_{\boldsymbol{\Theta}^{(t-1)},k}(\boldsymbol{x}_i)[\log\pi_k + \log f_k(\boldsymbol{x}_i;\boldsymbol{\Theta}_k)] - \mathcal{R}(\boldsymbol{\Theta}) \\
=& \max_{\boldsymbol{\Omega}_1,\ldots,\boldsymbol{\Omega}_K} \frac{1}{n}\sum_{i=1}^{n}\sum_{k=1}^{K} L_{\boldsymbol{\Theta}^{(t-1)},k}(\boldsymbol{x}_i)[\log\pi_k - \frac{p}{2}\log(2\pi) + \frac{1}{2}\log\det(\boldsymbol{\Omega}_k) \\
&-\frac{1}{2}(\boldsymbol{x}_i-\boldsymbol{\mu}_k)^\top\boldsymbol{\Omega}_k(\boldsymbol{x}_i-\boldsymbol{\mu}_k)] - \frac{1}{2}\mathcal{R}(\boldsymbol{\Theta}) \\
=& \max_{\boldsymbol{\Omega}_1,\ldots,\boldsymbol{\Omega}_K} \frac{1}{n}\sum_{k=1}^{K}\left\{\frac{1}{n}\sum_{i=1}^{n} L_{\boldsymbol{\Theta}^{(t-1)},k}(\boldsymbol{x}_i)[\log\det(\boldsymbol{\Omega}_k) - (\boldsymbol{x}_i-\boldsymbol{\mu}_k)^\top\boldsymbol{\Omega}_k(\boldsymbol{x}_i-\boldsymbol{\mu}_k)]\right\} - \mathcal{R}(\boldsymbol{\Theta}) \\
=& \max_{\boldsymbol{\Omega}_1,\ldots,\boldsymbol{\Omega}_K} \frac{1}{n}\sum_{k=1}^{K} n_k[\log\det(\boldsymbol{\Omega}_k) - \text{trace}(\widetilde{S}_k\boldsymbol{\Omega}_k)] - \mathcal{R}(\boldsymbol{\Theta}),
\end{aligned}$$

where the last equality is because

$$\begin{aligned}
&\frac{1}{n}\sum_{i=1}^{n} L_{\boldsymbol{\Theta}^{(t-1)},k}(\boldsymbol{x}_i)(\boldsymbol{x}_i-\boldsymbol{\mu}_k)^\top\boldsymbol{\Omega}_k(\boldsymbol{x}_i-\boldsymbol{\mu}_k) \\
=& \frac{1}{n}\sum_{\boldsymbol{x}_i\in\mathcal{A}_k} \text{trace}((\boldsymbol{x}_i-\boldsymbol{\mu}_k)(\boldsymbol{x}_i-\boldsymbol{\mu}_k)^\top\boldsymbol{\Omega}_k) \\
=& \frac{1}{n}\text{trace}\Big(\sum_{\boldsymbol{x}_i\in\mathcal{A}_k}(\boldsymbol{x}_i-\boldsymbol{\mu}_k)(\boldsymbol{x}_i-\boldsymbol{\mu}_k)^\top\boldsymbol{\Omega}_k\Big).
\end{aligned}$$

Then plugging in the last update of $\boldsymbol{\mu}_k$ leads to the desirable result. ■

Appendix C. Supporting Lemma

Lemma S.3 *Consider a finite number of independent centered sub-gaussian random variables X_i. Then $\sum_i X_i$ is also a centered sub-gaussian random variable. Moreover,*

$$\left\|\sum_i X_i\right\|_{\psi_2}^2 \le C\sum_i \|X_i\|_{\psi_2}^2,$$

where C is an absolute constant.

Lemma S.4 *Let X, Y be two sub-Gaussian random variables. Then $Z = X \cdot Y$ is sub-exponential random variable. Moreover, there exits constant C such that*

$$\|Z\|_{\psi_1} \le C\|X\|_{\psi_2}\cdot\|Y\|_{\psi_2}. \tag{S.39}$$

Lemma S.5 *Let X be sub-Gaussian random variable and Y be sub-exponential random variables. Then $X-\mathbb{E}[X]$ is also sub-Gaussian; $Y-\mathbb{E}[Y]$ is also sub-exponential. Moreover, we have*

$$\|X-\mathbb{E}[X]\|_{\psi_2} \le 2\|X\|_{\psi_2},\ \|Y-\mathbb{E}[Y]\|_{\psi_1} \le 2\|Y\|_{\psi_1}.$$

Lemma S.6 *Suppose $X_1, X_2, \ldots, X_n$ are n iid centered sub-Gaussian random variables with $\|X_1\|_{\psi_2} \leq K$. Then for every $t \geq 0$, we have*

$$\mathbb{P}\left(\left|\frac{1}{n}\sum_{i=1}^{n} X_i\right| \geq t\right) \geq e \cdot \exp\left(-\frac{Cnt^2}{K^2}\right),$$

where C is an absolute constant.

Lemma S.7 *Suppose $X_1, X_2, \ldots, X_n$ are n iid centered sub-expoential random variables with $\|X_1\|_{\psi_1} \leq K$. Then for every $t \geq 0$, we have*

$$\mathbb{P}\left(\left|\frac{1}{n}\sum_{i=1}^{n} X_i\right| \geq t\right) \geq 2 \cdot \exp\left(-C \min\left\{\frac{t^2}{K^2}, \frac{t}{K}\right\} n\right),$$

where C is an absolute constant.

Lemma S.8 *Hoeffding's inequality Suppose $X_1, X_2 \ldots X_n$ are independent random variable, $a_1 \leq X_i \leq b_i$, then we can have*

$$\mathbb{P}\left(\left|\frac{1}{n}\sum_{i=1}^{n} (X_i - \mathbb{E}X_i)\right| > \varepsilon\right) \leq 2 \exp\left\{\frac{-2n\varepsilon^2}{\frac{1}{n}\sum_{i=1}^{n}(b_i - a_i)^2}\right\}.$$

Moreover, if $a_i = 0$ and $b_i = 1$, then we have

$$\mathbb{P}\left(\left|\frac{1}{n}\sum_{i=1}^{n}(X_i - \mathbb{E}X_i)\right| > \varepsilon\right) > 1 - 2e^{-2n\varepsilon^2}.$$
